# The TP53-activated E3 ligase RNF144B is a tumour suppressor that prevents genomic instability

**DOI:** 10.1186/s13046-024-03045-4

**Published:** 2024-04-29

**Authors:** Etna Abad, Jérémy Sandoz, Gerard Romero, Ivan Zadra, Julia Urgel-Solas, Pablo Borredat, Savvas Kourtis, Laura Ortet, Carlos M. Martínez, Donate Weghorn, Sara Sdelci, Ana Janic

**Affiliations:** 1 Department of Medicine and Life Sciences, Universidad Pompeu Fabra, Barcelona, 08003 Spain; 2 Thoracic Cancers Translational Genomics Unit, Vall d’Hebron Institute of Oncology (VHIO), Barcelona, 08035 Spain; 3 Centre for Genomic Regulation (CRG), The Barcelona Institute of Science and Technology, Barcelona, 08003 Spain; 4 Pathology Platform, Instituto Murciano de Investigación Biosanitaria (IMIB-Pascual Parrilla), Murcia, 30120 Spain

**Keywords:** Cancer, Tumor suppressor, Genomic instability, Aneuploidy

## Abstract

**Background:**

TP53, the most frequently mutated gene in human cancers, orchestrates a complex transcriptional program crucial for cancer prevention. While certain TP53-dependent genes have been extensively studied, others, like the recently identified RNF144B, remained poorly understood. This E3 ubiquitin ligase has shown potent tumor suppressor activity in murine *Eμ Myc*-driven lymphoma, emphasizing its significance in the TP53 network. However, little is known about its targets and its role in cancer development, requiring further exploration. In this work, we investigate RNF144B's impact on tumor suppression beyond the hematopoietic compartment in human cancers.

**Methods:**

Employing TP53 wild-type cells, we generated models lacking RNF144B in both non-transformed and cancerous cells of human and mouse origin. By using proteomics, transcriptomics, and functional analysis, we assessed RNF144B's impact in cellular proliferation and transformation. Through in vitro and in vivo experiments, we explored proliferation, DNA repair, cell cycle control, mitotic progression, and treatment resistance. Findings were contrasted with clinical datasets and bioinformatics analysis.

**Results:**

Our research underscores RNF144B's pivotal role as a tumor suppressor, particularly in lung adenocarcinoma. In both human and mouse oncogene-expressing cells, RNF144B deficiency heightened cellular proliferation and transformation. Proteomic and transcriptomic analysis revealed RNF144B's novel function in mediating protein degradation associated with cell cycle progression, DNA damage response and genomic stability. RNF144B deficiency induced chromosomal instability, mitotic defects, and correlated with elevated aneuploidy and worse prognosis in human tumors. Furthermore, RNF144B-deficient lung adenocarcinoma cells exhibited resistance to cell cycle inhibitors that induce chromosomal instability.

**Conclusions:**

Supported by clinical data, our study suggests that RNF144B plays a pivotal role in maintaining genomic stability during tumor suppression.

**Graphical Abstract:**

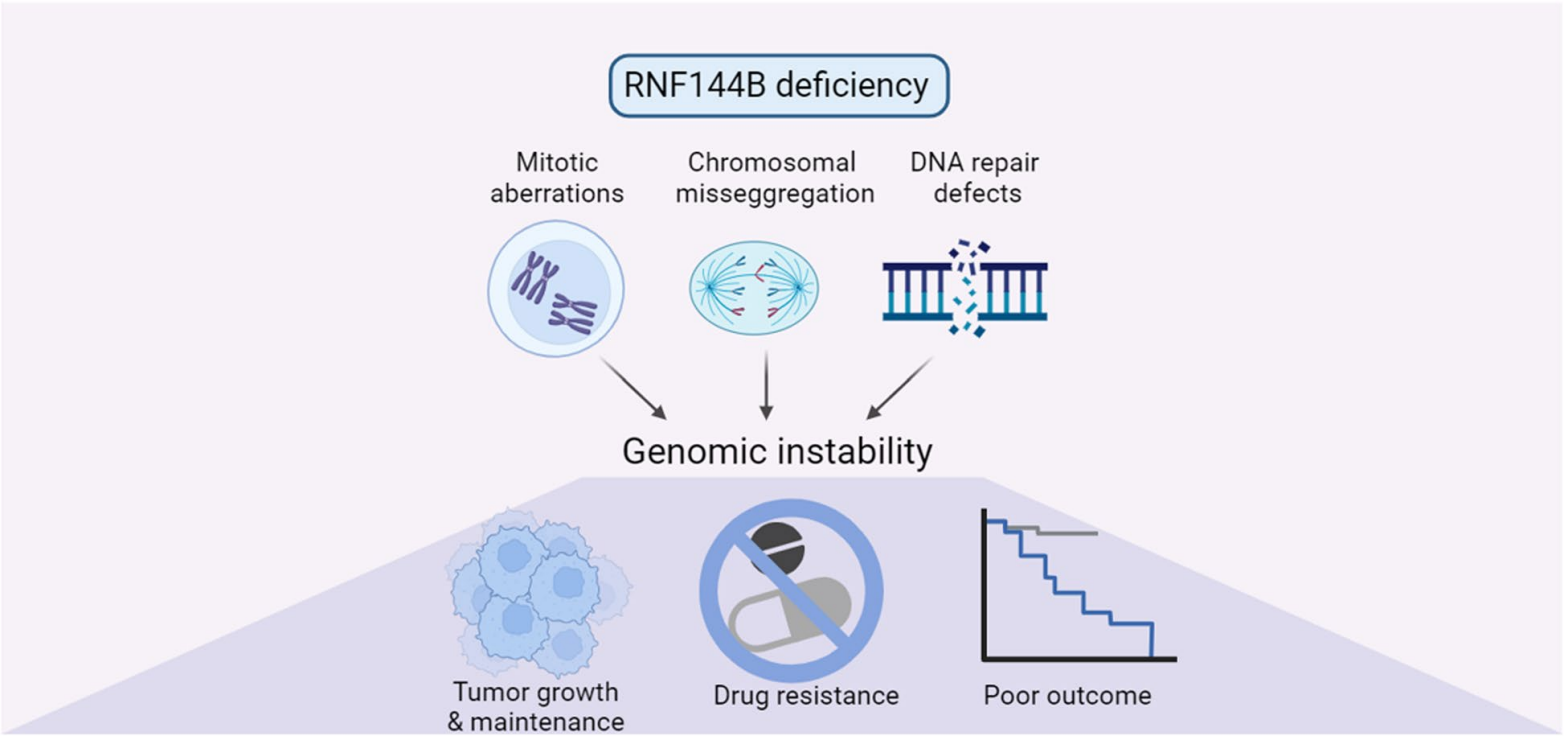

**Supplementary Information:**

The online version contains supplementary material available at 10.1186/s13046-024-03045-4.

## Background

*TP53* is recognized as the most frequently mutated gene in human cancers [1, 2]. Furthermore, germline heterozygous mutations in *TP53* cause the rare cancer predisposing Li-Fraumeni syndrome [3, 4] and 100% of mice lacking *Trp53* develop tumours, primarily lymphomas or sarcomas highlighting the pivotal role of TP53 as a tumour suppressor [5–7]. TP53 operates as a transcription factor, responding to a broad range of stress signals by binding to the DNA in a sequence-specific manner and activating many effector genes, ranging from several hundred to over a thousand [8–12]. These genes play crucial roles in multiple cell protective pathways, such as apoptosis, cell cycle arrest, senescence, DNA damage and repair mechanisms [8–10, 13]. Therefore, identifying the molecular mechanisms underlying TP53's function in tumour suppression is vital for understanding cancer development. Although some TP53-dependent genes, such as genes coding for the cell cycle inhibitor p21 or the pro-apoptotic proteins PUMA and Noxa, have been broadly studied in TP53-mediated tumour suppression [8, 14–19], numerous others still have an unknown relevance in the TP53 network. Several studies have uncovered the importance of such undervalued players of TP53-dependent tumour suppression, including ZMAT3 [20–23], ABCA1 [24], TIGAR [16] or GLS2 [25] among others. Recently, it has been described how the knock-down of several TP53-dependent proteins that have a role in DNA damage response, such as CAV1, MLH1, MSH2, DDIT4, POLK, ERCC5, FANCC or RNF144B, was enough to accelerate *Eμ Myc*-driven lymphoma [23, 26]. Remarkably, knockdown of *Rnf144b* substantially accelerated lymphoma development at a rate similar to knockdown of p53 itself. Moreover, mutations in RNF144B are largely mutually exclusive with mutations in p53 in several cancers, consistent with a notion that RNF144B and TP53 could function in the same pathway [23]. Importantly RNF144B role as a tumour suppressor in other cellular and oncogene driven contexts is still unknown.

RNF144B, also known as p53-inducible RING-finger protein (p53RFP), is an E3-ubiquitin ligase enzyme from the ubiquitin-ligases RBR (RING-in-between-RING) family [27–29] and is therefore partially involved in the proteasomal degradation of its targets by ubiquitin transference [28, 30]. RNF144A, homologous of RNF144B [28, 30], is a TP53-activated ubiquitin-ligase and it has been proposed as a tumour suppressor because it promotes proteasomal degradation of cytosolic DNA-PKc proteins and consequent apoptosis following DNA damage [31]. Previous studies have shown that RNF144B is strongly related to the TP53 family of transcription factors, including TP53 itself [23, 32, 33], TP63[34] and TP73 [35]. RNF144B regulates epithelial homeostasis and differentiation through degradation of the cell cycle inhibitor p21 [33] and modulates apoptosis [36, 37]. Due to its potentially important role as a tumour suppressor [23], it is important to investigate further the cellular functions of RNF144B and its role in TP53-mediated tumour progression.

Here, we investigate the role of RNF144B as a TP53-regulated tumour suppressor in different cellular and oncogenic contexts than *Eμ Myc*-driven lymphoma. Our studies coupled with in vivo, 3D or 2D cellular models’ analysis and clinical data, demonstrate that RNF144B suppresses cell proliferation and transformation, in particular in the context of lung cancer. Molecular analysis showed a novel function of RNF144B in maintaining genomic stability, resulting in effects on DNA repair and mitotic progression. Finally, RNF144B deficient cells gained resistance to cell cycle and chromosomal instability inducing drugs, commonly used in the clinics.

## Methods

### Cell culture

Human lung adenocarcinoma cell line A549 (ATCC, CRL-3588) and the colon carcinoma cell line HCT116 (ATCC, CCL-247) were obtained from Hospital del Mar Research Institute and authenticated using Short Tandem Repeat profiling (CSIC-UAM, Madrid, Spain). Mouse *KRAS*^*G12V*^ lung cancer cell lines (mKLC) were a gift from D. Santamaria (CIC, Spain) [38] and were grown in DMEM (L0102, Biowest) containing 10% Fetal Bovine Serum (FBS, S181BH, Biowest) and 100 µg/ml penicillin/ streptomycin (15,140,122, Gibco). HBEC3-KT (ATCC, CRL-4051) immortalized bronchial epithelial cells were a gift from Silvestre Vicent (CIMA, Spain) [39, 40]. HBEC3-KT cells were cultured in KSFM media (17,005,042, Gibco) containing 50 µg/mL of Bovine Pituitary Extract (BPE, 13,028,014, Gibco) and 5 ng/mL of human epidermal growth factor (hEGF, E9644, Gibco). HBEC3-KT cells expressing *KRAS*^*G12D*^ and sgRNA knockout populations were also cultured in RPMI-1640 (L0500, Biowest) media supplemented with 10% FBS and 100 µg/ml penicillin/ streptomycin. Mouse embryonic fibroblasts (MEFs) were generated from E13.5 C57BL/6J embryos. MEFs were grown in DMEM containing 10% FBS, 100 µg/ml penicillin/ streptomycin, 100 μM asparagine (A4159, Sigma) and 50 μM 2-mercaptoethanol (63,689, Sigma). Cells were grown in 5% CO_2_ at 37ºC. All cell lines were regularly tested for mycoplasma. Only mycoplasma-negative cells were used.

### Virus production and transduction

To generate CRISPR knockout bulk populations or clones, cell lines were transduced with a two-construct lentiviral pFUGW-derived system: a constitutive vector with an mCherry-labeled Cas9 [41] and a sgRNA expression vector [23] expressing CFP. sgRNAs sequences were cloned after BsmbI (R0580S, NEB) digestion. sgRNAs targeting human genes were the following: For human *Tp53*: 5’-GGCAGCTACGGTTTCCGTCT-3’, and for human *Rnf144b*: 5’-TGACATGGTGTGCCTAAACC-3’. A non-targeting control sgRNA was used (sgCTRL: 5’-CCAGTTGCTCTGGGGGAACA-3’).

shRNAs GFP-labeled targeting mouse RNF144B (shRNF144B: 5’-TGCTGTTGACAGTGAGCGCCAGGTTATTTACATACTTTCATAGTGAAGCCACAGATGTATGAAAGTATGTAAATAACCTGATGCCTACTGCCTCGGA-3’), TRP53 (5’-TGCTGTTGACAGTGAGCGCCCACTACAAGTACATGTGTAATAGTGAAGCCACAGATGTATTACACATGTACTTGTAGTGGATGCCTACTGCCTCGGA-3’) or the shRenilla control (5’TGCTGTTGACAGTGAGCGCAGGAATTATAATGCTTATCTATAGTGAAGCCACAGATGTATAGATAAGCATTATAATTCCTATGCCTACTGCCTCGGA-3’) were generated into LMS (LTR/MCSV/SV40-puro-IRES-GFP) retroviral vector [23]. 3KT cells were infected with a Lenti-CMV-KRAS^G12D^ construct [39]. To immortalize MEFs cell cultures, retroviral vectors expressing *E1a* and *Hras*^*G12V*^ were used [23]. For the in vivo competition assay, control MEFs were transduced with a lentiviral plex-Renilla-mCherry (gift from Dr A. Celià-Terrassa, Hospital del Mar Research Institute, Spain). To perform drug response analysis of live cells, a construct expressing a nuclear localization signal (NLS) coupled to GFP was used: pTRIP-SFFV-EGFP-NLS (NLS-GFP) was a gift from Nicolas Manel (Addgene plasmid # 86677). For the overexpression studies, RNF144B cDNA construct (NM_182757.4) was generated in a pcDNA3.1( +)-C-6His vector (Genscript, Netherlands).

Lentiviral supernatant was generated by transient transfection of HEK293T (ATCC, CRL-3216) cells with the following packaging constructs: pMDL (5 μg), pRSV-rev (2.5 μg) and pVSV-G (3 μg)[23]. For retroviral particle production GAG (4.8 µg), and pENV (2.4 µg) constructs were used [23]. 10 μg of vector DNA was transfected using calcium phosphate precipitation. Viral supernatant from HEK293T cells was collected after 48 h, filtered, transferred to cell cultures, and centrifuged at 2200 rpm at 32ºC during 2 h. After 48-72 h, cells were FACS-sorted for the corresponding fluorescence using a BD Influx cell sorter (BD Biosciences). If needed, CRISPR single cell clones were seeded in 96 well plates and expanded to generate isogenic populations. MEFs infected with *E1a* and *Hras*^*G12V*^ were selected with Puromycin (3 μg/ml, P7255, Sigma) and Hygromycin (200 μg/ml, 400,052, Sigma) for 72 h. MEF immortalized cell lines were used at low passage (passage 6–14) to avoid phenotypes arising from prolonged passaging.

### Animal experiments

All animal experiments are compliant with ethical regulations regarding animal research and were conducted under the approval of the Ethics Committee for Animal Experiments (CEEA-PRBB, Barcelona, Spain). All animals were euthanized before or at the moment of achieving maximum tumour volume. Subcutaneous tumour models were performed by injection of 1 million cells suspended in 100 μl of PBS in both flanks of 7–10-week-old female Athymic Nude-Foxn1nu mice (Envigo). Tumours were grown for approximately 3 weeks and harvested at the endpoint. For in vivo competition assay, MEF cells were infected with the plex-Renilla-mCherry lentiviral construct or with GFP-labeled shRNA targeting RNF144B or TRP53. Cells were mixed 1:1, evaluated by FACS (LSR Fortessa, BD Biosciences) and 1 million cells were injected subcutaneously into the flanks. After 3 weeks tumours were harvested, minced, and digested in a solution of DMEM, 0,3 mg/ml Collagenase I (C1-BIOC, Sigma) and 10 μg/ml DNAse I (DN25, Sigma) at 37ºC while shaking for 2 h. Digested tumours were filtered through a 45 μM mesh, cleaned of red blood cells with Red Blood Lysis Buffer (11,814,389,001, Roche) and analyzed by cytometry (Fortessa). Subcutaneous tumour growth was followed by caliper measurements and the following formula applied to measure tumour volume: volume = 1/2(length × width^2^). In the case that tumours did not grow in the flank, measurement was excluded from the comparative analysis.

Intercostal intrapulmonary model was performed by injecting 200.000 A549 cells suspended in 10 μl of PBS through the ribcage into the left lung with a 29G insulin needle and a depth of 4–4.5 mm. 10–12-week-old female Athymic Nude-Foxn1nu mice were used for this study. Weight was monitored biweekly, and animals were euthanized at 6 weeks post-inoculation. Only mice with localized intrapulmonar tumours were considered for tumour burden analysis.

3KT experiments were performed by injecting 1 million cells in 100ul PBS intravenously in the tail vein and after 5 months, animals were euthanized to study lung lesions.

Lungs were inflated with 4% paraformaldehyde (PFA, sc-281692, SCBT) through the trachea and fixed overnight for histological evaluation. Lung sections were performed and scanned with an Aperio ScanScope (Leica) at the Anatomy Department (Hospital del Mar). Tumour area and lung area were measured with ImageJ to calculate tumour burden. Those mice that didn’t present any tumour growth, or that had tumoural growth outside the lung and into the thoracic space were excluded from the analysis. Mice were housed in groups of 5 per cage and irradiated chow and water were provided ad libitum.

### Proliferation analysis

50.000 3KT cells were seeded in 6 well plates in triplicates and after 6 days of growth, cells were counted using Trypan Blue staining and a Countess 3 Automatic cell counter (Thermofisher). The experiment was performed in five independent replicas.

### Colony formation assay

3.000 3KT cells were seeded in 6 well plates and after 8 days of growth, cells were fixed using 4% PFA for 10 min and stained with 0.5% crystal violet solution (V5265, Sigma-Aldrich) for 1 h. Plates were scanned with an Amersham Typhoon™ (Cytiva). Crystal violet was dissolved with 10% acetic acid and absorbance was read at 590 nm in a Biotek Synergy HTXmachine (Agilent). The experiment was performed in four independent replicas.

### Spheroid cultures

1.000 3KT cells were resuspended in 50 μl of cold Matrigel GFR (354,230, Corning) and seeded as a drop in the wells of a 24 well plate. Soon after seeding the Matrigel domes, the plate was turned upside down and placed in the incubator for 30 min. Afterwards, 1 ml of KSFM media with 20% FBS and 1% penicillin/ streptomycin was added. Spheroids were monitored for 7 days and pictures were taken using a brightfield Olympus CKX53 microscope and an Olympus EP50 camera. Pictures were taken of 4–5 random fields per well with a 10 × objective. Spheroid diameter was analyzed by ImageJ. The number of spheroids quantified was between 180 and 410 depending on the cell line. Experiment was repeated twice and performed in three technical replicates each time.

### Immunoblotting

Cells were lysed in RIPA buffer containing protease inhibitors (cOmplete protease inhibitor cocktail, 11,836,170,001, Roche). Protein extracts were quantified using the Protein Assay Dye Reagent (5,000,006, BioRad) and 20 μg were separated by SDS-PAGE and transferred onto nitrocellulose membranes (Cytiva Amersham). Membranes were blocked for 1 h in 5% milk in PBS-T (PBS with 0.1% Tween 20) and incubated overnight with the corresponding primary antibody in PBS-T 5% milk. For probing antibodies against TRP53 (NCL-L-p53-CM5p, Leica Biosystems), TP53 (sc-126, SCBT), p-γH2AX (9718 T, CST), βACTIN (sc-47778, SCBT), His-Tag (66,005–1-Ig, ThermoFisher), and secondary antibodies anti-rabbit (sc2357, SCBT), and anti-mouse (sc-516102, SCBT) were used. Membranes were developed using the ECL Prime system (RPN2232, Cytiva) and imaged using a ChemiDoc MP (BioRad).

### Overexpression analysis

2,5 × 10^5^ A549 or 5 × 10^5^ 3KT cells were seeded in 6 well plates. 24 h after seeding were transfected with 1500 ng of the empty vector (pcDNA3.1 + C-6His) or the OE-RNF144B vector (RNF144B_OHu07981C_pcDNA3.1( +)-C-6His) using Lipofectamine 2000 reagent (11,668,027, ThermoFisher) following manufacturer instructions. Cells were counted after 72 h using Trypan Blue staining and the automatic cell counter Countess 3. A pellet of cells was collected to perform western blot and confirm overexpression. Experiment was repeated thrice and performed in triplicates.

### Immunohistochemistry

Tissues were collected and fixed in 4% PFA overnight and processed for paraffin-embedding. Slides were stained for Hematoxylin and Eosin (H&E) using standard protocols. Immunohistochemistry was performed with antibodies against Ki67 (12202s, CST) and pH3 (3377 T, CST). Briefly, paraffin sections were re-hydrated and antigen retrieval was performed in a pressure cooker with Sodium Citrate Buffer pH6 for 20 min. 3% H_2_O_2_ was used to quench the peroxidase for 15 min and blocking was done with PBS / BSA 1% (A9647, Sigma) / 0,3 Triton-X (11,332,481,001, Sigma) for 30 min. Slides were incubated overnight at 4ºC in a humid chamber with primary antibody. The next day, sections were incubated with the 2º antibody (Impress HRP Goat Anti-Rabbit, MP-7451–15, Vector Laboratories) for 1.30 h and afterwards incubated with DAB peroxidase kit (K346711-2, Agilent) and hematoxylin. Slides were mounted with DPX mounting media (06522 Sigma). A Cell Observer (Zeiss) microscope was used for imaging. Images were analyzed and quantified using Qupath [42] (v0.3.2).

### Amplicon sequencing of sgRNA target sites

A549 (isogenic clones) and 3KT cells (bulk population) carrying Cas9 and sgNT or sgRNF144B were evaluated by amplicon sequencing to detect INDELs in the sgRNA target site. Genomic DNA was extracted from the cells using the DNeasy Blood and Tissue kit (69,504, Qiagen). The sgRNA target sites were PCR amplified using primers flanking the site of interest with recommended overhangs (Fwd:5’-ACACTCTTTCCCTACACGACGCTCTT CCGATCTGTGGCTGAAATGTGTGAGCA-3’ and Rev: 5’-GACTGGAGTTCAGACGTG TGCTCTTCCGATCTCTGTATTTTCTTGCTAGACTCC-3’). PCR was performed to ensure a single band was amplified and PCR products were purified using the QIAquick PCR Purification Kit (28,104, Qiagen) and sent to Genewiz (Leipzig, Germany) using Amplicon-EZ service, able to read from 150-500 bp.

### qRT-PCR

Cells were treated with 10 μM Nutlin-3a for 6 h to stimulate TP53 activation or left untreated, depending on experiment. Total RNA was isolated from cells using TRIzol reagent (15,596,018, ThermoFisher) and reverse transcribed using SuperScript III (18,080,400, ThermoFisher) or SuperScript IV (18,090,050, ThermoFisher), Reverse Transcriptase and Oligo-d(T) primers (18,418,020, ThermoFisher). qRT–PCR was performed using either SYBR green (Roche, 4,707,516,001) or Taqman Gene Expression assays (ThermoFisher). For Taqman: Human TP53 (Hs01034249_m1), mouse TRP53 (Mm01731287_m1), human CDKN1A (Hs00355782_m1), mouse CDKN1A (Mm00432448_m1), human RNF144B (Hs00403456_m1), mouse RNF144B (Mm00461356_m1), housekeeping human HMBS (Hs00609297_m1) and mouse HMBS (Mm01143545_m1). For SYBR green the primers were as follows: Human RNF144B: 5´-TTGTCCTGCCAACAGAGCAC-3´ and human GAPDH: 5´-GCACAGTCAAGGCCGAGAAT-3´. Samples were analyzed in QuantStudio 12 K equipment (Applied Biosystems). The mRNA expression levels of TP53 target genes of interest were standardized with corresponding housekeeping genes and normalized to the untreated control.

### Metaphase spread

1,5 million cells were seeded in 10cm^2^ plates and treated the following day with 0.3 mg/ml of colcemid (10,295,892,001, Roche) for 3 h. Cells were collected and resuspended dropwise with KCL 0.056 M and incubated during 20 min at RT. Cells were then fixed in cold methanol:glacial acetic acid solution (3:1) and washed 3 more times with the fixative solution. Cells were dropped on glass slides from 1,5 m height, dried and stained with 3% Giemsa (GS500, Sigma). After washing, coverslips were mounted and pictures were captured using a brightfield Olympus CKX53 microscope and an Olympus EP50 camera, using a 40 × objective. Chromosomes were counted manually with ImageJ Software. At least 25 cells were analyzed per cell line/genotype.

### Cell cycle assay

450.000 cells were seeded in 6 well plates and the next day, cells were trypsinized, washed with PBS and fixed with cold ethanol (70%) in a dropwise manner while vortexing. After 2 h of fixation, cells were pelleted, washed twice with PBS and resuspended in working solution, containing 15 μg/ml of Propidium Iodide (00–6990-50, ThermoFisher) and 300 μg/ml RNAse A (10,109,142,001, Sigma). Cells were incubated for 2 h at room temperature (RT) and cell cycle distribution was analyzed by flow cytometry using a BD LSRII-B cytometer (BD Biosciences). Data was analyzed using FlowJo software.

### Edu incorporation

Edu incorporation was performed using the Click-iT EdU Alexa Fluor 647 Flow Cytometry Assay Kit (C10424, ThermoFisher). Between 250.000–350.000 cells were seeded in 12 well plates and pulsed with 10 μM EdU for 2 h. Next, cells were harvested, fixed, permeabilized and stained using the Click-iT EdU Alexa Fluor 647 Flow Cytometry Assay Kit following manufacturer’s instructions. Cells were co-stained with a solution containing Propidium Iodide (15 μg/ml) and RNAse (300 μg/ml) to measure DNA content. Samples were analyzed using BD LSRII-B cytometer and FlowJo Software.

### DNA repair quantification by immunofluorescence

15.000 cells were seeded in Phenoplate (6,055,302, PerkinElmer) black well plates and the following day cells were gamma-irradiated at 5 Gy with an IBL-437C (CIS Biointernational). Control plate was left untreated. Cells were fixed with 4% PFA. Afterwards, blocking and permeabilization was performed with PBS/5% BSA/0,3% Triton-X during 1 h at RT. Staining with the primary antibody p-γH2AX (9718 T, CST) dissolved in PBS/1% BSA/0,3% Triton-X was performed overnight at 4ºC. The following day, cells were washed × 3 with PBS and secondary anti-rabbit Alexa Fluor 647 (A21244, Invitrogen) was added during 2 h at RT in the dark. Cells were washed again × 2 with PBS and incubated with 1 μg/ml DAPI (D9542, Sigma) for 10 min. After washing, cells were imaged with the Opera or Operetta High Content Screening System (Perkin Elmer), using the 40 × objective. Segmentation of the nuclei using the DAPI signal and quantification of the number of p-γH2AX foci per cell was done using Harmony® High-Content Imaging and Analysis Software.

### Cell viability assays

1 × 10^5^ MEFs, A549 and 3KT cells were seeded in a 24-well flat bottom plate in medium containing 10% FCS. 24 h after seeding, the cells were incubated with Doxorubicin (0.05 μg/ml or 0.2 μg/ml), Nutlin-3a (10 μM) or with 0% FBS media, respectively. For 0% FBS experiments, cells were washed 3 × with PBS to remove any residual FBS before addition of medium. Cells were harvested 24 h or 72 h after, stained with APC Annexin V kit (640,920, Biolegend) and 1 μg/ml DAPI and analyzed with an BD LSRII-B cytometer and FlowJo Software.

### Immunofluorescence imaging of mitotic cells

Between 120.000 and 150.000 cells were grown in 24-well plates. The day after cells were treated or not with 15 µM RO-3306 for 18 h at 37 °C, 5% CO_2_. Cells were washed with PBS, fixed with 4% PFA for 10 min at RT and permeabilized with PBS / 0.1% Triton X-100 for 5 min at RT. Blocking (RT, 20 min) and incubations with antibodies (RT, 1 h) were performed with 10% FBS in PBS 0.1% / Triton X-100 and washes were done with PBS 0.1% / Triton X-100 at RT for 3 × 5 min. The antibodies targeted α-tubulin (T9026, Sigma) and γ-tubulin (T6557, Sigma). An Alexa 555 Goat anti-Mouse antibody (A-21424, Invitrogen) was used as a secondary antibody. Nuclei were counterstained with 1 μg/mL DAPI for 2 min at RT and cells were mounted using the ProLong Gold antifade reagent (P10144, Thermofisher). Confocal microscopy pictures were taken with a Leica STELLARIS microscope. For counting lagging chromosomes, DNA bridges, multipolar mitosis or centrosome numbers, at least 200 cells were analyzed by eye for each condition.

### Micronuclei assay

150.000 cells were grown in 24-well plates. The day after, cells were washed with PBS and fixed in freshly prepared 4% PFA for 10 min at RT. Nuclei were counterstained with 1 μg/mL DAPI in PBS for 2 min at RT and cells were mounted using the ProLong Gold antifade reagent. Confocal microscopy pictures were acquired in a z-stack mode with a Leica STELLARIS microscope. Micronuclei analysis has been made with Fiji software and for each field (45 random field/sample) the number of micronuclei were divided by the number of nuclei.

### Live cell imaging of mitotic cells

100.000 cells were grown on 4-well chambered coverslips (80,426, Ibidi). The day after, cells were treated with 15 µM RO-3306 for 18 h. One hour before imaging, siR-Hoechst (SC007, Spirochrome) was added to the media at 1 µM and cells were incubated at 37 °C and 5% CO_2_. Just before imaging, media was replaced by FluoroBrite DMEM (A1896701, ThermoFisher) supplemented with 10% FBS and siR-Hoechst. Time-lapse live-cell imaging was performed using a Leica STELLARIS confocal system with white light laser inverted microscope maintaining temperature at 37 °C and CO_2_ at 5%. Images were taken every 4 min with a × 64 objective. Exposure time was optimized so that no phototoxicity or photobleaching was caused to cells. Image processing was performed using FIJI software.

### In vitro cell growth assay

A549 and 3KT cells expressing Cas9 and sgNT, sgRNF144B or sgTP53 were infected with the NLS-GFP construct and sorted for GFP + cells. 5 × 10^3^ cells were seeded in 96 Phenoplate black well plates. 24 h after seeding were treated with Palbociclib (1–3 μM, 3 μM, Hospital del Mar), Abemaciclib (0,5–3 μM, Hospital del Mar), Paclitaxel (10–20 nM, S1150, Selleckchem), Docetaxel (5–20 nM, Hospital del Mar), Etoposide (10–20 μM, 341,205, Sigma), Doxorubicin (0,05–0,2 μg/ml, N31815, Sigma), Carboplatin (50–100 μM, Hospital del Mar), RO-3306 (5 μM, HY-12529, MedChem) and Nutlin-3a (20 μM). Imaging was performed as described previously [43] with the Operetta High Content Screening System using the × 20 magnification. Cell number represented by the sum of the nuclear GFP intensity/well was quantified with the Harmony Software at day 0 (prior to drug treatment) and after 48 or 72 h, depending on the cell line. Cell confluency was normalized to that of day 0 of the same well.

### LC–MS/MS Proteomics and analysis

MEFs were infected with the corresponding shRNAs in three independent biological replicas and sorted for GFP by flow cytometry using a BD Influx cell sorter (BD Biosciences). Afterwards, cells were washed with PBS, scrapped with 6 M Urea and 200 mM Ammonium Bicarbonate and sonicated at 4ºC. 10 μl of each sample at 1 mg/ml was submitted for analysis. The samples were digested with Trypsin and LysC and 2 μg were analyzed by LC–MS/MS using a 90 min gradient in the Orbitrap Eclipse. Raw MS files were processed in Proteome Discoverer version 2.3.0.523 (Thermo Scientific, Waltham, MA,) [44]. Samples have been searched against SP_Mouse database (June 2020), using the search algorithm Mascot v2.6 (http://www.matrixscience.com/). Peptides have been filtered based on FDR and only peptides showing an FDR lower than 5% have been retained. Normalized protein abundances with “Total Peptide Amount” from Proteome Discoverer were used as input for the analysis with the DEP R package [45].

6513 quantified protein profiles were expressed on 9 samples. We only kept proteins that were based at least in two unique peptides, leading to a final protein quantification data matrix of 5389 proteins. Proteins with missing values showed a lower expression in reference to those without missing values. A full normalized matrix of protein expression values was obtained by imputing missing quantifications with a mixed methodology. Proteins with missing at random (MAR) values were imputed with k-nearest neighbors (knn) algorithm and missing not at random (MNAR) values were imputed with random draws from a Gaussian distribution centered around a minimal value (MinProb). We conducted a protein differential expression analysis based on protein-wise linear models and empirical Bayes statistics using limma [46]. Proteins with *p*-value < 0.05 and a minimum fold-change of 50% were considered as statistically significant. 5039 proteins had no significant change in expression while 350 proteins were differentially expressed between the control replicates and the RNF144B knockdown cells.

Functional enrichment analysis of the biological processes was conducted with the Gene Ontology (GO) database using the clusterProfiler package [47]. Significant GO terms are shown with an associated *p*-adjusted value (determined by circle color) and GeneRatio (Number of differentially abundant proteins associated with the GO terms / number of input differentially abundant proteins). The circle size is given by the count of proteins detected that are involved in each GO term.

### RNA-Seq analysis

MEFs were infected with the corresponding shRNAs in three independent biological replicas and sorted for GFP with a BD Influx cell sorter. After, cells were trypsinized and the pellet was snap frozen. RNA from 1 million cells was extracted using the Purelink RNA kit (10,307,963, Invitrogen) and submitted for analysis. Libraries were prepared using the TruSeq stranded mRNA Library Prep (20,020,594, Illumina) according to the manufacturer's protocol. Briefly, 1000–500 ng of total RNA were used for poly(A)-mRNA selection using poly-T oligo attached magnetic beads using two rounds of purification. RNA was fragmented under elevated temperature and primed with random hexamers for cDNA synthesis. Then, cDNA was synthesized using reverse transcriptase (SuperScript II, 18,064–014, Invitrogen) and random primers. The addition of Actinomycin D to the First Strand Synthesis Act D mix (FSA) prevents spurious DNA-dependent synthesis, improving strand specificity. After that, second strand cDNA was synthesized, incorporating dUTP in place of dTTP to generate ds cDNA using DNA Polymerase I and RNase H. A corresponding single T nucleotide on the 3’ end of the adapter provided a complementary overhang for ligating the adapter to the fragments. It was followed by subsequent ligation of the multiple indexing adapter to the ends of the ds cDNA. Finally, PCR was performed with a PCR Primer Cocktail. Final libraries were analyzed using Bioanalyzer DNA 1000 or Fragment Analyzer Standard Sensitivity (DNF-473, Agilent), and were then quantified by qPCR using the KAPA Library Quantification Kit KK4835 (07960204001, Roche) prior to the amplification with Illumina’s cBot. Libraries were sequenced 1 * 50 + 8 bp on Illumina's HiSeq2500.

We performed a quality control on the 9 raw single-end reads samples using the nf-core/rnaseq (*v. 3.10.1*) [48, 49]. Raw FASTQ files were aligned to the GRCm38.p6 version of the reference mouse genome using STAR (*v. 2.7.6a*) [50] with default parameters except for –sjdbOverhang 49, producing a set of 9 BAM files. Aligned reads in BAM files were reduced to a table of 55,487 genes by 9 samples. Genes were annotated using the GENCODE vM25 GTF file. Following previously established recommendations [51, 52], we filtered out lowly expressed genes by discarding those that did not show a minimum reliable level of expression of 20 counts per million reads of the smallest library size, in at least all the samples of the smallest group, which was 3. After the filtering, we ended up with a final table of counts of 14,668 genes by 9 samples. The DESeq2 package (*v. 1.40.0*) [52, 53] was used for the differential expression analysis. Surrogate variables were calculated with SVA [54]. Genes with adjusted p-value < 0.05 (5% FDR) and absolute log2FC > 1 were considered as statistically significant.

Integrative transcriptomics vs proteomics analysis was conducted to show the expression relationship patterns of differentially expressed genes vs differentially abundant proteins. Results are represented with Log_2_ expression ratio. The cut-offs are a minimum fold-change of 50% for the proteomics expression profile and minimum fold-change of 100% for the transcriptomics expression profile.

### RNF144B differential expression study

To access comprehensive data on GTEx, GDC and TCGA Pan-Cancer normalized gene expression, phenotypic information, and somatic mutations, we utilized XenaBrowser [55] to extract publicly available data from The Cancer Genome Atlas (TCGA) (https://www.cancer.gov/tcga). The combined cohort of TCGA, and GTEx [56] samples were employed to investigate gene expression differences between normal and tumour samples. To study RNF144B expression in *Tp53* wild type or mutated tumors, the GDC-TCGA Pancancer dataset was utilized. Samples were stratified depending on their classification as *Tp53* wild type or mutant and *Tp53* was considered wild type in the following conditions: no mutation present, synonymous variants (silent) or located in the intronic, 5' UTR, or 3' UTR regions. *Tp53* was considered mutant in the following conditions: splice mutations, frameshift, stop codon gain, missense mutation, coding sequence variants, inframe insertions and loss of start or stop point mutations. The considerations for *Tp53* status stratification and the specific mutations present in the patient samples are shown in Supplementary Table 1. When analyzing RNF144B expression data, we focused on cancer types that contained a minimum of 20 samples, with both *Tp53* wild type and *Tp53* mutated entries present in the gene expression matrix. The cancer types that didn’t reach the minimum 20 samples per group are: Ovarian (OV), uterine (UCEC), testicular (TGCT), papillary kidney (KIRP), cervical (CESC), thymus (THYM), mesothelioma (MESO), skin melanoma (SKCM), bile duct (CHOL), clear cell kidney (KIRC), thyroid (THCA), myeloid leukemia (LAML), rectum (READ), B-cell lymphoma (DLBC), uterine (UCS), adrenocortical (ACC), pheocromocytoma (PCPG) and uveal melanoma (UVM) malignancies. Unpaired two-tailed t-test was conducted to evaluate the statistical differences in log-normalized read counts of RNF144B between tissues or cancer types. In order to facilitate visual comparison across TCGA datasets with wild type or mutant *Tp53*, the expression of RNF144B was mean centered to zero prior to plotting.

GDC-TCGA Pancan datasets were analyzed with www.xenabrowser.net for Kaplan Meyer analysis. Samples were stratified by *Tp53* status, and by the gene expression levels of RNF144B (being high expression those samples with normalized expression values equal or above the median value and low expression the lower half). Samples containing null data were excluded. Kaplan Meier plots for 10-year overall survival were plotted for remaining samples. Comparison between groups was evaluated with a log rank test.

Analysis of RNF144B as a *Tp53* target gene in different mouse and human databases was performed using the TargetGeneRegulation database [57].

### CERES effect

RNF144B dependencies in human lung cancer cell lines were analyzed using the Achilles DepMap dataset (DepMap Public 22Q4 + Score, Chronos) [58]. Cell lines were categorized in *Tp53* mutant or *Tp53* wild type and the CERES dependency score was plotted for each of them.

### ChIP-sequencing data analysis

To perform the analysis of ChIP-seq data, the FASTQ files were acquired from the Sequence Read Archive (SRA) within the Gene Expression Omnibus (GEO) public repository. The specific accession numbers GSE71175 [59] and GSE55727 [60] were utilized to retrieve the FASTQ files corresponding to mouse and human cells, respectively. We used the nf-core/chipseq pipeline (*v. 1.2.2*) [48, 49]. FASTQ reads were aligned to the GRCm38.p6 reference genome. MACS2 [61] was used to call peaks in the narrowPeak mode. Peaks with a q-value < 10e-5 were considered statistically significant. The resulting data was visualized using the Gviz R package [62].

### Aneuploidy scores analysis

For the assessment of aneuploidy scores, we utilized the gene expression dataset from the TCGA Pan-Cancer (PANCAN) cohort. Aneuploidy scores were directly obtained from [63]. RNF144B log-normalized read counts were stratified in high and low expression using the median as cut-off. Samples were also stratified by TP53 status, following the criteria used in TCGA Pan-Cancer dataset. The term "PANCAN" denotes the analysis across all cancer types together.

### GSEA analysis

GSEA [64] was carried out in R using the fgsea package v1.24.0 [65], using as a gene set the list of 70 genes (CIN70) with the highest levels of consistent correlation with ‘total functional aneuploidy’ (tFA) from [66]. The C2 curated collection from the Molecular Signatures Database (MSigB) portal was associated with the CIN70 signature. GSEA was used to test for enrichment of specific gene sets within a ranked list based on *p*-value and log_2_FC to define whether the chromosomal instability profile is enriched among the overexpressed proteins of our analysis.

## Results

### RNF144B plays a role within the TP53 pathway in several human cancers

**Fig. 1:**
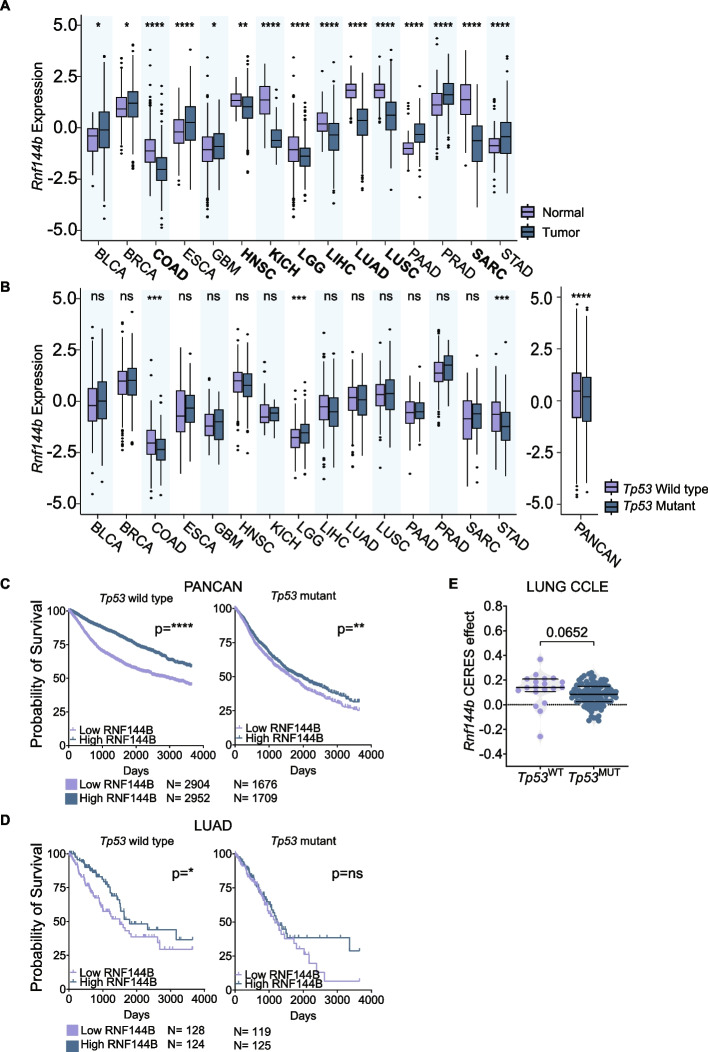
RNF144B is regulated by TP53 in different contexts. **A** Normalised RNF144B mRNA expression in healthy (light blue) and primary tumour (dark blue) samples across 15 different cancer types from GTEx and TCGA datasets. In bold, tumour types where RNF144B expression is significantly reduced in tumour compared to normal tissue: colon (COAD), head and neck (HNSC), kidney (KICH), brain (LGG), liver (LIHC), lung (LUAD and LUSC) and soft tissue (SARC) cancers. ****P ≤ 0.0001; **P ≤ 0.01; *P ≤ 0.05, *p* -values, two-tailed t-test. **B** Normalised RNF144B expression in TP53-proficient (light blue) and TP53-deficient (dark blue) tumour samples across 15 different cancer types and Pancancer analysis from GDC-TCGA Pancancer dataset. ****P ≤ 0.0001, ***P ≤ 0.01, *p*-values, two-tailed t-test. **C** and **D** Probability of ten-year overall survival of cancer patients in human TCGA (**C**) Pan-Cancer or (**D**) LUAD samples with TP53 wild-type or TP53 mutant status and RNF144B low (below the median) or high expression (above the median). ****P ≤ 0.0001; **P ≤ 0.01; *P ≤ 0.05, *p*-values, log-rank test. **E** Project Achilles lung cancer cell lines from CCLE dataset were segregated by TP53 status (wild-type or mutant) and plotted by CERES effect upon RNF144B depletion. *P*-value, two-tailed t-test

RNF144B has been described as a potent tumour suppressor in mouse B cell lymphoma models [23]. To test if RNF144B could have a tumour suppressor activity in human cancers, we analyzed its expression in publicly available datasets of cancer and healthy tissues (XenaBrowser [55]). We found that RNF144B expression was both downregulated or upregulated in different tumour tissues when compared to normal tissues, respectively (Fig. 1A), indicating its context-dependent regulation. To evaluate if RNF144B expression is TP53 dependent, we classified tumour tissue samples from GDC and TCGA datasets as TP53-deficient and TP53-proficient, based on their TP53 status (Supplementary Table 1). Pan-Cancer analysis revealed that RNF144B expression was significantly lower in the TP53-deficient versus the TP53-proficient tumours (Fig. 1B). Several TP53-deficient tumors, including colon, head and neck, liver, lung adenocarcinoma and stomach cancers showed lower expression levels of RNF144B in comparison to TP53-proficient tumour samples, however this association only reached statistical significance in colon and stomach cancer datasets (Fig. 1B). These correlative studies suggest that RNF144B could have a tumour suppressive role in certain cancer types, particularly when TP53 is wild-type.

The TP53-dependent expression of RNF144B in various human cancer types prompted us to address if RNF144B expression is associated with disease outcome. Human GDC and TCGA Pan-Cancer analysis revealed that disease-survival of the patients with low RNF144B expression was significantly reduced compared to those with higher RNF144B expression, independently of their TP53 status (Fig. 1C). Interestingly, when analysing this correlation across multiple cancer types (Fig. S1), we observed that decreased expression of RNF144B significantly correlated with a worsened prognosis exclusively in the LUAD patients with wild-type TP53, and not in those with mutant TP53 (Fig. 1D), supporting the role for RNF144B in LUAD, particularly when TP53 wild-type is functional.

To further examine the role of RNF144B in lung cancers we analysed lung cancer cell lines data from Project Achilles [58] and found that RNF144B inactivation enhances proliferation of lung cancer cells, especially those with wild-type TP53 (Fig. 1E).

Together, insights from human cancer analyses suggest potential tumor suppressor role of RNF144B in lung adenocarcinoma.

### TP53 regulates the expression of RNF144B in diverse cellular contexts

Given that human cancer data indicated the importance of RNF144B in tumor suppression in lung context, we sought to interrogate the role of RNF144B using lung adenocarcinoma models. TP53 plays a key tumor suppressive role in LUAD, with nearly 50% of tumors carrying TP53 mutations [67]. Moreover, our analysis of RNF144B expression and patient prognosis indicates potential regulation of RNF144B by TP53 in LUAD cancer. To establish controlled cell platforms to assess the expression of RNF144B in TP53-dependent manner and its impact in cellular phenotypes, we used TP53 wild type non-transformed and tumour-derived lung cell lines, and their TP53-deficient derivatives. Of note, the non-transformed (but immortalised) cell models we used maintain naturally near diploid characteristics and have limited and defined genetic alterations.

**Fig. 2:**
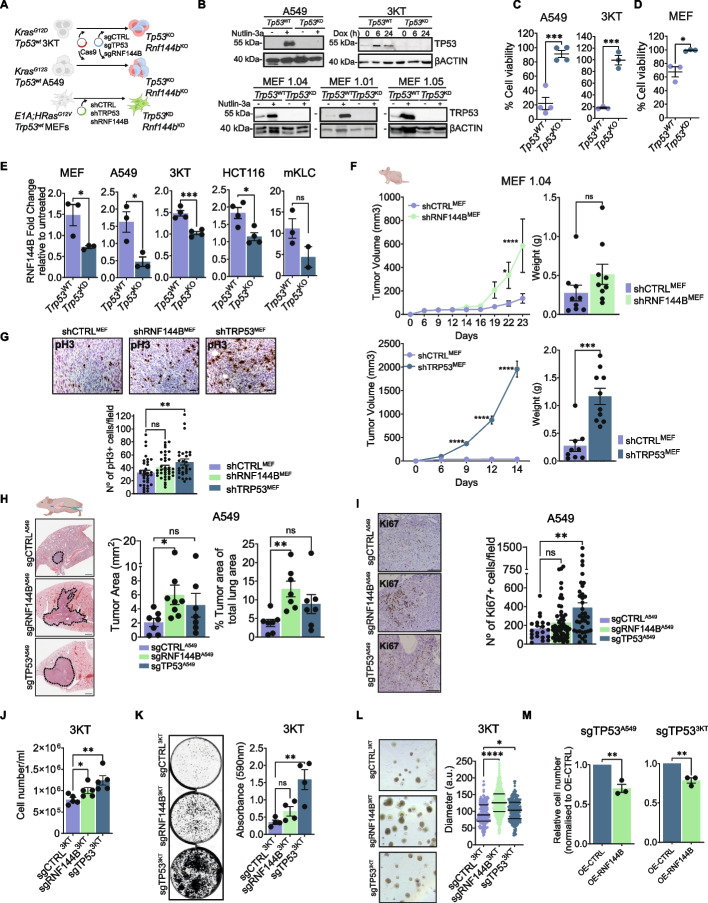
RNF144B suppresses oncogene expressing cell proliferation and transformation. **A** Schematic representation showing the generation of non-transformed (immortalised) MEFs and human bronchial epithelial cells (3KT), and tumour derived human A549 LUAD cells with three different variants: TP53 WT, TP53 deficient and RNF144B deficient. **B** Western blot analysis of A549, 3KT and MEFs cell line derivatives showing TP53 expression upon 6 h of treatment with MDM2 inhibitor, nutlin-3a (10 μM) or after 6h and 24h of treatment with doxorubicin (0.2 μg/ml), activators of TP53. Note that TP53 is expressed at low levels in non-treated TP53^WT^ control and treated TP53^KO^ or TP53^KD^ cell line derivatives, respectively. Probing for β-ACTIN was used as a loading control. **C** Human GFP-NLS tagged A549 and 3KT cell lines derivatives with two different TP53 states, wild type or deficient, were treated with Nutlin-3a (10 μM) for 72 h. Cell viability was measured by measuring nuclear GFP signal of images acquired with the Operetta High Content Screening System in confocal mode. GFP quantification was normalised to the respective untreated control. Data are presented as Mean ± SEM. for a minimum of 1300 cells. *N* = 3 or 4 independent experiments. *P*-values, ***P < 0.001; two-tailed student’s t-test. **D** The TP53 WT or deficient MEFs were treated with Nutlin-3a (10 μM) for 24 h. Cell viability was measured by staining cells with Annexin V plus DAPI followed by flow cytometric analysis. Annexin V- DAPI- cells were regarded as live cells. Data are presented as Mean ± SEM. *N* = 3 independent experiments *P*-value *P ≤ 0.05, two-tailed t-test. **E** qRT-PCR analysis of RNF144B mRNA expression in TP53 proficient and TP53 deficient MEFs, A549, 3KT, HCT116 and mKLC cells upon 6 h treatment with Nutlin-3a relative to untreated cells of the same genotype. *N* = 3–4 independent experiments for each cell line and cell variant, in duplicates. Data are presented as Mean ± SEM, *P*-values ***P ≤ 0.001; *P ≤ 0.05, two-tailed unpaired t-test. **F** MEF 1.04 cell line that has been transduced with indicated shRNAs were injected subcutaneously into nude mice and tumor volume was measured over 20 days. (Left) Tumour volume (mm3) of the same genotype (Right) Tumour weight at ethical endpoint. *N* = 9–10 tumours/shRNA from one MEFs cell line (1.04). Data are presented as Mean ± SEM. ****P ≤ 0.0001; ***P ≤ 0.001; *P ≤ 0.05. *p*-values, two-way or one-way ANOVA, respectively. **G** H&E staining and immunohistochemistry of pH3 at ethical endpoint, in particular at 15 days for shTRP53 and 24 days for shCTRL and shRNF144B MEF tumours. (Above) Representative images. Scale bar = 50 μm. (Below) Quantification of pH3 + cells. *N* = 5–6 tumours/shRNA. Total of 40 fields/shRNA were quantified. Data are presented as Mean ± SEM, **P ≤ 0.01, one-way ANOVA. **H** H&E-stained lung sections from mice 6 weeks after inoculation with A549 LUAD cells that have been transduced with indicated sgRNAs. (Left) Representative images. Scale bar = 800 μm. (Middle) Quantification of tumour area. (Right) Quantification of tumour area (%) relative to lung area. *N* = 7–8 tumours/sgRNA. Data are presented as Mean ± SEM. **P ≤ 0.01; *P ≤ 0.05; one-way ANOVA. **I** H&E staining and immunohistochemistry of Ki67 detected in mice 6 weeks after intrapulmonary injection with A549 LUAD cells that have been transduced with indicated sgRNAs. (Left) Representative images. Scale bar 130 μm. (Right) Quantification of Ki67 + cells. Total of 20–55 fields/sgRNA were quantified. *N* = 7–8 tumours/sgRNA. Data are presented as Mean ± SEM. **P ≤ 0.001, one-way ANOVA. **J** Cellular proliferation assay in 3KT cells transduced with indicated sgRNAs over 6 days. *N* = 5 independent experiments for each cell variant, in triplicates. Data are presented as Mean ± SEM, *P*-value **P ≤ 0.01; *P ≤ 0.05, one-way ANOVA. **K** Low-density plating assay in 3KT cells transduced with indicated sgRNAs over 8 days after seeding. (Left) Representative images after crystal violet staining. (Right) Quantification of the crystal violet intensity. *N* = 4 independent experiments for each cell variant, in triplicates. Mean ± SEM, *P*-value **P ≤ 0.01, one-way ANOVA. **L** Matrigel-embedded spheroids derived from 3KT cells transduced with indicated sgRNAs over 7 days. (Left) Representative images. (Right) Quantification of spheroid diameter. 400–700 spheroids/sgRNA were quantified, *N* = 2 independent experiments for each cell variant, in triplicates. Mean ± SEM. ****P ≤ 0.0001, *P ≤ 0.05, one-way ANOVA. **M** Cellular proliferation assay in TP53 deficient A549 or 3KT cells transduced with overexpression (OE) vectors, empty (CTRL) or expressing full-length Rnf144b. Bar graph represents the cell number relative to the CTRL, respectively. *N* = 3 independent experiments for each cell variant, in triplicates. Mean ± SEM. **P ≤ 0.01, two-tailed unpaired t-test

To investigate the role of RNF144B we used CRISPR-Cas9-mediated gene editing in *Kras*-driven lung models. We selected the HBEC3-KT cell line derived from human normal lung bronchial epithelia (hereafter 3KT) [40]. 3KT cells were transduced with *KRAS*^*G12D*^-expressing lentivirus and then infected with Cas9 and sgRNAs targeting TP53 or non-targeting control, to establish polyclonal TP53 wild type (sgCTRL^3KT^) and TP53 null (sgTP53^3KT^) human normal lung bronchial epithelial cell lines (Fig. 2A). As a tumour derived cell line, we selected human A549, a lung adenocarcinoma cell line derived from Type II alveolar epithelium expressing TP53 wild type and *KRAS*^*G12S*^. Using CRISPR/Cas9-mediated gene editing we generated TP53 null (sgTP53^A549^) and control (sgCTRL^A549^) isogenic A549 cell lines (Fig. 2A). In addition to lung cancer cell lines, we used primary mouse embryonic fibroblasts (hereafter MEF) cell lines expressing *E1A* and *HRas*^*G12V*^ oncogenes. These cells serve as a widely used cellular model with an intact TRP53 signaling pathway, where TRP53 plays a critical role in tumor suppression [68]. For this, three independent early-passaged *E1A* and *HRas*^*G12V*^ oncogene-expressing MEFs, were transduced with shRNAs targeting *Trp53* (shTRP53^MEF^) or a control shRNA targeting Renilla luciferase (shCTRL^MEF^) (Fig. 2A). Western blot analysis of the engineered cell lines confirmed the efficient removal or knockdown of the wild type TP53 protein levels (Fig. 2B). Quantitative RT-PCR (qRT-PCR) analysis of p21 expression, canonical target of TP53 [69, 70], confirmed the abrogation of TP53 signaling in the isogenic TP53 knockout cellular models (Fig. S2A), as well as their resistance to the MDM2 inhibitor Nutlin-3a, a potent activator of TP53 (Fig. 2C, D).

To test the notion that *Rnf114b* is a TP53 target gene, we examined the expression of *Rnf114b* in various cell types. We showed that *Rnf144b* expression is induced in TP53-dependent fashion in TP53 wild type MEFs, A549, and 3KT cells in response to Nutlin-3a (Fig. 2E). In addition, *Rnf144b* was also induced in other cell types, such as well characterised mouse TP53 wild type *KRAS*^*G12V*^-driven lung adenocarcinoma cells (mKLCs) [38] and human HCT116 colorectal cancer cells [71] (Fig. 2E and Fig. S2A and B). These findings demonstrate that TP53 can control the expression of RNF144B in certain mouse and human cellular contexts.

To assess deeper RNF144B regulation by TP53 we analysed chromatin immunoprecipitation sequencing (ChIP-seq) using previously published mouse [59] and human [60] datasets. We confirmed that the RNF144B locus is directly bound by TP53, both in human (Fig. S2C) and mouse cells (Fig. S2D). The observed peak in the human sample dataset corresponded with the p53 target sequence identified as p53BS1, which was already characterized to be present in the promoter region of RNF144B in a human glioblastoma cell line [33].

Finally, we consulted the TargetGeneReg database which contains detailed information on the network of genes that are regulated by TP53 [57]. As expected, p21 achieves a perfect "TP53 Expression Score" of 57 (within a range of -55 to 57), indicating consistent and widespread TP53 regulation. RNF144B obtains a score of 31, with 32 out of 57 human datasets analyzed showing a positive correlation between TP53 activation and RNF144B expression, and only 1 dataset showing a negative correlation. (Fig. S2E, Supplementary Table 2). A similar pattern was obtained for TP53 *bona fide* targets such as Noxa [14, 16], PTPN14 [72], or SLC43A2 [73] (32, 12, and 34 datasets, respectively), underscoring the context-specific nature of TP53 regulation. Regarding mouse datasets, the correlation was not as robust, as TP53 activation correlated with increased RNF144B expression in just 4 out of 15 datasets (Fig. S2E, Supplementary Table 3). Altogether, these results suggest that RNF144B could be directly regulated by TP53 in different contexts.

### RNF144B suppresses proliferation and transformation in lung epithelial cells and MEFs

To test whether RNF144B itself has growth suppressor capacity in vivo we transduced three independent oncogene-expressing *E1A* and *HRas*^*G12V*^ MEF lines with retroviruses expressing shRNAs against RNF144B (shRNF144B^MEF^) (Fig. 2A). We confirmed the RNF144B knockdown in MEFs by qRT-PCR (Fig. S3A). Analysis of these cells demonstrated that attenuated expression of RNF144B protein increased tumour growth in vivo upon subcutaneous injection into immunocompromised mice (Fig. 2F and Fig. S3B). shRNF144B^MEF^ subcutaneous tumours showed no differences in the level of phospho-Histone H3 (pH3, a proliferation marker) compared to control shCTRL^MEF^, while shTRP53^MEF^ tumours exhibited a significant increase in pH3 staining (Fig. 2G) indicative of more aggressive tumours. We further quantitatively examined the growth advantage of shRNF144B^MEF^ relative to control shCTRL^MEF^ by performing in vivo competition assay. We mixed 1:1 mCherry expressing shCTRL^MEF^ and GFP expressing shRNF144B^MEF^ or shTRP53^MEF^ and injected the cells subcutaneously into recipient immunocompromised mice (Fig. S3C and Supplementary Table 4.1 and 4.2). GFP-labeled shRNF144B^MEF^ and shTRP53^MEF^ dominated the resulting subcutaneous tumours, validating the in vivo growth advantage caused by RNF144B knockdown.

To further investigate the role of RNF144B in oncogene driven tumour growth, we selected a A549 LUAD model, in which RNF144B is induced by TP53 (Fig. 2E). By CRISPR/Cas9-mediated gene editing we generated an isogenic A549 cell line lacking full-length RNF144B protein (sgRNF144B^A549^) (Fig. 2A). The efficiency of RNF144B knock-out was confirmed by qRT-PCR (Fig. S3D) and amplicon sequencing of the targeted gene region (Fig. S3E). Interestingly, our results showed that sgRNF144B^A549^ cells had enhanced tumour growth upon orthotopic intrapulmonary injection into immunocompromised mice, displaying a significant increase in tumour size and tumor burden (Fig. 2H). We observed no significant differences between the RNF144B^A549^ and control tumours in the proliferation index, as determined by Ki67 staining (Fig. 2I). In contrast, TP53^A549^ displayed increased numbers of Ki67 positive nuclei, indicative of more aggressive adenocarcinoma lesions (Fig. 2I), consistent with the crucial tumor suppressive function of p53 in mutant KRAS-driven LUAD.

To evaluate whether RNF144B plays a role in lung adenocarcinoma growth we knockout RNF144B in non-transformed *KRAS*^*G12D*^ expressing lung bronchial 3KT epithelial cell line by CRISPR/Cas9 (sgRNF144B^3KT^) (Fig. 2A). RNF144B knock-out efficiency was confirmed by qRT-PCR (Fig. S3D) and amplicon sequencing (Fig. S3E). Combined overexpression of *KRAS*^G12D^ and knockout of RFN144B enhanced growth of 3KT cells in 2D and 3D cultures compared with control sgCTRL^3KT^ cells (Fig. 2J-L), highlighting its potency in lung cancer growth and transformation. Interestingly, upon intravenous injection into immunocompromised mice, sgRNF144B^3KT^ cells developed lung adenocarcinoma lesions, while these lesions were not evident in control sgCTRL^3KT^ mice (Fig. S3F). Next, we tested the impact of enforced RNF144B expression on cell proliferation driven by the loss of TP53 in 3KT cells and A549 cells. TP53-deficient sgTP53^A549^ and sgTP53^3KT^ cells transduced with a vector encoding RNF144B had reduced proliferation capacity compared to control cells (Fig. 2M and Fig. S3G). These findings show that RNF144B can impede the growth of the human LUAD cells.

Taken together, oncogene-expressing cells lacking RNF144B gained proliferation and transformation capacity, in particular MEF and lung cells. We next examined apoptosis in response to two different insults: acute DNA damage and serum starvation. In agreement with previously published data in *EμMyc*-overexpressing B cells [23], we showed that RNF144B has no impact on cell death in oncogene expressing MEF, 3KT and A549 cellular models (Fig. S3H). We next sought to understand which additional cellular processes might be also regulated by RNF144B during transformation suppression by performing various molecular and cellular assays.

### RNF144B regulates expression of proteins involved in mitotic progression and DNA damage response

**Fig. 3:**
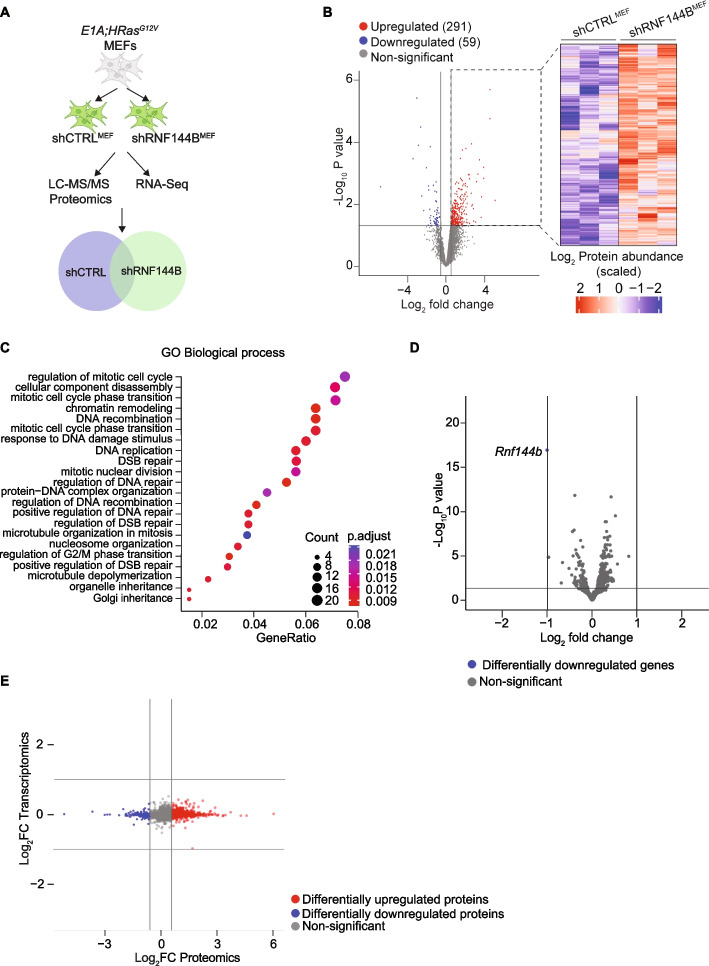
Cellular proteome is altered upon RNF144B knockdown. **A** Schematic representation of LC–MS/MS proteomics and RNA-Seq experiment. **B** (Left) Volcano plot of shRNF144B^MEF^ compared to shCTRL^MEF^ proteins. Red proteins are significantly increased in abundance, and blue are significantly reduced. In gray, not significant. (Right) Heatmap of differentially abundant proteins in shRNF144B^MEF^ and shCTRL^MEF^.**C** GO biological processes associated with significantly altered proteins in shRNF144B^MEF^ versus shCTRL^MEF^. **D** Volcano plot of RNA-Seq transcriptomics analysis of shRNF144B^MEF^ compared to shCTRL^MEF^. In blue, Rnf144b transcript was significantly reduced as expected. In gray, not significant transcripts. **E** Integrative representation of transcriptomics and proteomics analysis of shRNF144B^MEF^ versus shCTRL^MEF^. Red proteins are enriched in the proteomic datasets, blue are depleted in the proteomic dataset and not significantly altered at the transcriptome level. In gray, non-significant changes at proteomics or transcriptomic level. *N* = 3 replicas/shRNA

To gain mechanistic insights into how RNF144B suppresses cellular proliferation and transformation, we sought to identify the molecular players involved in RNF144B directed pathways. RNF144B contains a conserved RING-between-RING domain and possesses E3 ubiquitin ligase activity [27–29] and therefore can participate in the targeting and proteasomal degradation of other proteins by ubiquitin transfer. To identify molecular pathways regulated by RNF144B we performed steady-state proteomics in low passaged oncogene-expressing shRNF144B^MEF^ and shCTRL^MEF^ (Fig. 3A). Direct comparison of shRNF144B^MEF^ with shCTRL^MEF^ cells revealed differentially abundant proteins associated with cell cycle (p21, TRP53, Top2a, Cdk2), chromatin remodeling (Baz1b, H1-1), DNA damage repair and microtubule organization (TPX2, POLB, BCL7C, RAD21) (Fig. 3B-C, Fig S4A, Table S5). Among 291 differentially upregulated proteins, TRP53 appeared elevated in shRNF144B^MEF^ (Fig. S4A), prompting us to validate steady-state levels of TRP53 protein levels in various cell lines. While TP53 levels were consistently elevated in a relatively narrow range within shRNF144B^MEFs^, the large variations in TP53 levels were observed between individual experiments within sgRNF144B^3KT^ and no differences in sgRNF144B^A549^ LUAD cancer cells (Fig. S4B). This indicates that TP53-regulation is likely independent of RNF144B or could occur at tissue- or stage- specific manner.

In parallel, we performed RNA-seq analysis to examine whether the detected changes in protein levels upon RNF144B knockdown were due to the underlying differences in mRNA levels. As expected, considering the E3 ubiquitin ligase function of RNF144B, the downregulation of RNF144B did not provoke major alterations at the transcriptomic levels (Fig. 3D and Supplementary Table 5). Integrative analysis of our transcriptomic and proteomic data showed that most of the changes occurred at the proteomic level, with a clear trend towards upregulation of protein abundance in shRNF144B^MEF^ cells (Fig. 3E). Together, these results suggest that RNF144B may control the degradation of proteins related to cell cycle progression, microtubule organisation and DNA damage response during transformation suppression.

### RNF144B regulates ploidy maintenance and DNA damage response

**Fig. 4:**
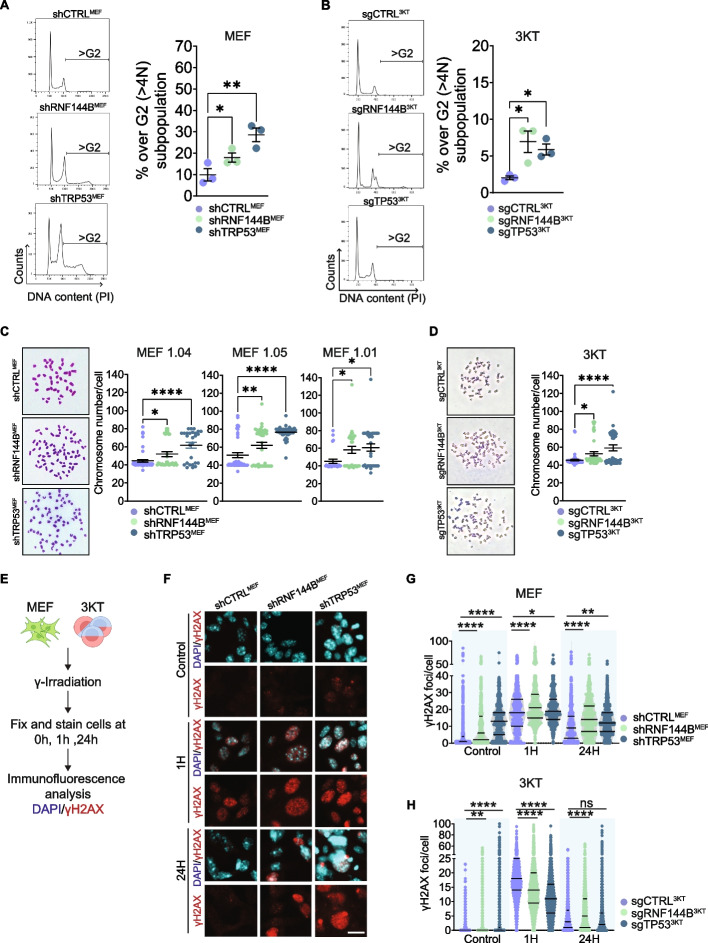
RNF144B deficiency leads to aneuploidy and increased DNA damage susceptibility. DNA content measured by propidium iodide flow cytometry analysis in (**A**) MEF 1.04 and (**B**) 3KT cell lines transduced with indicated shRNAs or sgRNAs, respectively. (Left) Representative histogram profiles. (Right) DNA content quantification for profiling more than 4N (aneuploid) cells (> G2). *N* = 3 independent biological replicates per cell line derivative. **C** (Left) Representative metaphase spreads from MEFs transduced with indicated shRNAs. (Right) Quantification of chromosome number per cell from metaphase spreads. *N* = 3 cell lines/shRNAs. Minimum of 25 cells were analyzed per cell line/shRNA. **D** Metaphase spreads from 3KT transduced with indicated sgRNAs. (Left) Representative pictures. (Right) Quantification of chromosomes per cell. Minimum of 45 cells were analyzed per cell line/sgRNA. **E** Schematic representation of MEFs and 3KT cells irradiated, fixed at different timepoints (0 h, 1 h and 24 h) and images taken at 40X magnification. **F** Representative pictures of γH2AX foci in MEF 1.04 cell line transduced with different shRNAs, in different timepoints after IR. DAPI marks nuclei. Scale bar: 20 μm (**G**) Quantification of γH2AX foci per cell in MEF 1.04 cell line with *N* = 570–1300 cells analysed per shRNA/timepoint in two independent experiments. **H** Quantification of γH2AX foci per cell in 3KT cell line with *N* = 2274–5105 cells analysed per shRNA/timepoint in two independent experiments. Data are presented as Mean ± SEM. ****P ≤ 0.0001; **P ≤ 0.01, *P ≤ 0.05, one-way ANOVA

Proteomics results prompted us to further investigate the role of RNF144B in controlling cell cycle progression and DNA repair, processes implicated in cellular transformation suppression. Analysis of DNA content showed that RNF144B deficient MEFs and 3KT lung cells displayed a subpopulation (> G2) (Fig. 4A and B and Fig. S5) that has features of aneuploidy (DNA content > 4n), similar to TP53 deficient cells (positive control), but absent in control TP53 proficient cells (Fig. 4A and B and Fig. S5).

To further investigate karyotype abnormalities, we conducted chromosome spread analysis, and revealed that RNF144B deficiency in both oncogene-expressing MEF and lung 3KT cells exhibited a greater proportion of cells with elevated chromosome numbers compared to control cells, similar to TP53 deficient cells (Fig. 4C and D).

Beyond maintaining genomic integrity by regulating ploidy, RNF144B has been shown to promote DNA repair in *EμMyc*-overexpressing B cells [23]. Interestingly, the capacity of RNF144B to regulate DNA repair has been supported by our proteomics analysis (Fig. 3C and Fig. S4A). To determine whether RNF144B function is linked to DNA repair in oncogene-expressing cells we conducted immunofluorescence analysis with γH2AX (a marker for double strand breaks) both in RNF144B deficient MEFs and 3KT before and after γ-Irradiation (IR) (Fig. 4E). Absence of RNF144B led to significantly higher levels of γH2AX foci in the basal state and late response to DNA damage (0 h and 24 h after IR), in both MEFs and 3KT cells (Fig. 4F-H), similar to TP53 deficient cells**.** These findings suggest that RNF144B plays an important role in maintaining ploidy and supporting DNA damage response in non-transformed oncogene expressing cells.

### RNF144B deficiency drives chromosomal instability

**Fig. 5:**
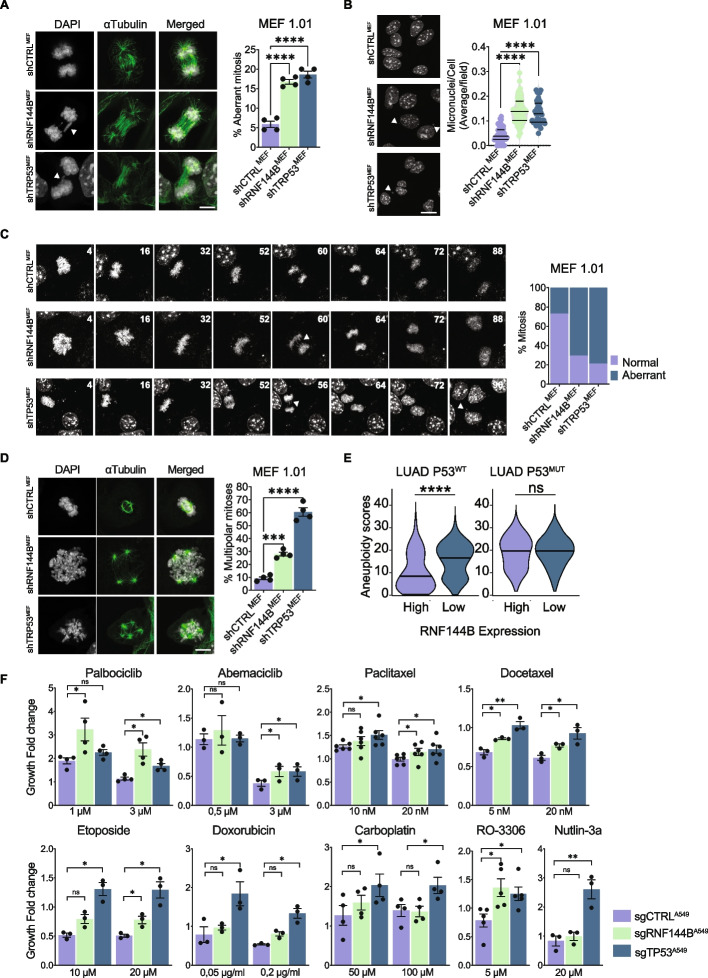
RNF144B deficiency triggers chromosomal instability during mitosis. **A** Mitotic analysis. (Left) Representative anaphase images of α-tubulin and DAPI staining in MEF 1.01 cell line transduced with indicated shRNAs. Arrows show lagging chromosomes. Scale bar: 10 μm. (Right) Quantification of aberrant mitosis containing lagging chromosomes or DNA bridges. At least 800 mitoses (the same proportion of the different phases of mitosis) per cell line were analyzed in two independent experiments; in duplicates, each dot represents a replicate. Data presented as mean ± SEM ****P ≤ 0.0001, one-way ANOVA. **B** Micronucleus count by DAPI staining in MEF 1.01 cell line transduced with indicated shRNAs. (Left) Representative images of micronuclei. Arrows show micronuclei. Scale bar: 10 μm. (Right) Quantification of micronuclei. At least 45 fields were analyzed per condition in two independent experiments, each dot represents a field. Data presented as mean ± SEM ****P ≤ 0.0001, one-way ANOVA. **C** (Right) Live-cell time-lapse representative pictures of MEF 1.01 cell line after release from 15 μM RO-3306-induced G2 arrest. transduced with indicated shRNAs, stained with siR-Hoechst (not all pictures are represented here). White arrows show mitotic aberrations, like lagging chromosomes or micronuclei appearing post mitosis. (Left) Quantification of aberrant mitosis. *N* = 17–38 mitosis/shRNA were quantified for analysis. **D** Mitotic pole analysis of MEF 1.01 cell line transduced with indicated shRNAs, uponrelease from 15 μM RO-3306-induced G2 arrest. (Left) Representative images of α-tubulin and DAPI staining. Scale bar: 10 μm (Right) Quantification of multipolar mitosis. At least 400 mitoses/shRNA were analyzed in two independent experiments, in duplicates, each dot represents a replicate. Data presented as mean ± SEM ****P ≤ 0.0001; ***P ≤ 0.001, one-way ANOVA. **E** Violin plot of the tumour aneuploidy scores in high versus low RNF144B mRNA expression in LUAD cohort, separated by TP53 status. ****P ≤ 0.0001, two-tailed t-test. **F** In vitro growth of GFP-NLS tagged A549 LUAD cells transduced with indicated sgRNAs and treated for 72 h with a panel of cytotoxic drugs: Palbociclib (1 μM, 3 μM), Abemaciclib (0,5 μM, 3 μM), Paclitaxel (10 nM, 20 nM), Docetaxel (5 nM, 20 nM), Etoposide (10 μM, 20 μM), Doxorubicin (0,05 μg/ml, 0,2 μg/ml), Carboplatin (50 μM, 100 μM), RO-3306 (5 μM) and Nutlin-3a (20 μM). Cell growth was measured by measuring nuclear GFP signal of images acquired with the Operetta High Content Screening System in confocal mode. Cell growth was normalized to day 0 of the same well/genotype. At least 1000 cells/well were analysed in 3–5 independent experiments, in duplicates. **P ≤ 0.01; *P ≤ 0.05, paired t-test student

Aneuploidy and DNA damage have been proposed to be a product of chromosomal instability (CIN) in cancer cells [74]. We sought to determine whether RNF144B downregulation triggers hallmarks of large-scale CIN, such as lagging chromosomes, anaphase bridges, multipolar mitosis or micronuclei, in unstressed oncogene-expressing MEFs. In mitotic shRNF144B^MEF^ cells, the number of lagging chromosomes or DNA bridges was significantly increased than in control shCTRL^MEF^ cells (Fig. 5A). Analyses of the micronuclei content resulted in a significantly increased frequency of cells containing micronuclei in shRNF144B^MEF^, that could be attributed to missegregated chromosomes (Fig. 5B). We observed significant differences in the number of centrosomes and multipolar mitosis in non-stressed shTRP53^MEF^, but comparatively fewer in shRNF144B^MEF^ and shCTRL^MEF^ (Fig. S6A and B). To confirm that aneuploidy in shRNF144B^MEFs^ cells results from defects in chromosome segregation, we performed time-lapse video and immunofluorescence analysis of mitotic oncogene-expressing MEFs cells released after a temporary G2-arrest by RO-3306 (G2/M inhibitor) and found that shRNF144B^MEF^ have significantly elevated proportion of cells with DNA bridges/lagging chromosomes (Fig. 5C, Fig. S6C and Movie S1) and multipolar mitosis (Fig. 5D and Movie S2). As expected, the positive control shTRP53^MEF^ cells displayed significantly higher frequency of cells with lagging chromosomes or DNA bridges, micronuclei, multipolar mitosis and an increased number of centrosomes in comparison to control shCTRL^MEF^ (Fig. 5A-D, Fig. S6A-C and Movies S3, 4 and 5). Collectively, these findings indicated the occurrence of severe chromosomal aberrations, confirming that downregulation of RNF144B triggers hallmarks of large-scale genomic instability due to mitotic failures.

The analysis of aneuploidy scores in human TCGA datasets indicated that low mRNA expression of RNF144B correlated with higher aneuploidy score in LUAD patients with TP53 wild type while this correlation was not significant when testing for TP53 mutant LUAD tumours (Fig. 5E). The negative correlation of RNF144B expression and aneuploidy showed to be significant too when human TCGA Pan-Cancer data were analyzed (Fig. S5D). In addition, we have observed correlation between the RNF144B regulated protein signatures (Fig. 3B and Fig. S4A) with a previously described chromosomal instability signature [66] (Fig. S6E), supporting the link between the genomic instability and RNF144B molecular axis.

All together, we found that RNF144B is a tumour suppressor involved in maintaining genomic stability and its knockdown triggers the appearance of several mitotic defects that eventually lead to chromosomal aberrations and DNA damage in oncogene expressing cells.

### RNF144B deficient cancer cells are resistant to drugs that target cell cycle and cause chromosomal instability

Our results support the notion that RNF144B plays a significant role in the DNA damage response, mitosis progression and chromosomal instability. These could indicate that cancer cells that are deficient for RNF144B have reduced sensitivity to drugs that specifically target the cell cycle, CIN or induce DNA damage. To test this hypothesis, we tested a panel of cytotoxic agents in A549 and 3KT cells lacking RNF144B. We used different families of drugs, including microtubule stabilizers (paclitaxel, docetaxel), CDK4/6 inhibitors that cause prolonged arrest cells in G1/S (palbociclib, abemaciclib), CDK1 inhibitor that promotes G2/M arrest (RO-3306), DNA damaging topoisomerase II inhibitors (etoposide, doxorubicin) and an intercalating DNA agent (carboplatin). Nutlin-3a was used as a positive control for the cell viability assays. Cells were treated with increasing concentrations of drugs and analysed for cell growth. Interestingly, RNF144B deficiency led to significant protection to CIN-inducing [75, 76] cell cycle inhibitors Palbociclib and Abemaciclib, as well as to microtubule stabilizers Paclitaxel and Docetaxel, and the DNA damaging agent Etoposide in A549 and 3KT cell lines. As anticipated, positive control TP53 deficient cells were resistant to the majority of the drugs tested, while A549 and 3KT transduced with control sgRNAs were sensitive to the treatments (Figs. 5F and S7A). We next observed that RNF144B deficient cells had elevated levels of γH2AX following Etoposide, Doxorubicin, Paclitaxel and Docetaxel treatment, which was particularly evident in the A549 LUAD cells, indicating that chemoresistance could be caused due to increased tolerance to the presence of DNA damage. While, treatment with Palbociclib, Abemaciclib, and RO-3306 did not induce DNA lesions, as shown by the absence of γH2AX (Fig. S7B). Many reports indicate that the status of p53 determines the cellular response to cytotoxic agents [77]. However, our results show that p53 is not the sole determinant because RNF144B deficient 3KT and A549 cells, both have different p53 levels yet demonstrated quite similar responses to cytotoxic agents.

Collectively, TP53-activated target RNF144B plays a crucial role in maintaining the genomic stability by controlling the degradation of multiple proteins involved in mitotic progression and DNA damage. Decreased RNF144B levels are associated with heightened aneuploidy in human tumors and can ultimately impact the effectiveness of drugs that target the cell cycle and induce chromosomal instability in human lung adenocarcinoma cancer.

## Discussion

TP53, a crucial tumour suppressor, regulates diverse cellular processes, including apoptotic cell death, cell cycle arrest and senescence, giving rise to distinct mechanisms proposed for TP53-mediated tumor suppression in different contexts [8–10, 13]. Through an in vivo genetic screening in hematopoietic stem/progenitor cells, RING Finger Protein RNF144B was identified as a critical factor contributing to TRP53-mediated tumor suppression, in the context of blood cancers [23]. We focused on better understanding RNF144B, which has been a poorly characterized tumor suppressor identified as one of the most significant hits in the screen. Here we show that RNF144B deficiency enhances growth of epithelial non-transformed and tumour derived cells, in particular lung cells, and its enforced expression is capable of inhibiting lung cancer cell proliferation driven by TP53 loss. The growth-suppressive role for RNF144B in human LUAD cancers, particularly when TP53 is intact, has been further emphasized by our human cancer genome analysis, that indicate tight correlation between TP53 status, RNF144B expression, and the prognosis of the LUAD patients. This highlights the pivotal role of RNF144B in cancer cell proliferation and transformation across various contexts.

Previous studies have shown that RNF144B is an E3-ubiquitin ligase enzyme [27–29] and is therefore involved in the proteasomal degradation of its targets. Here, to understand RNF144B function we employed a proteomic and RNA-sequencing analysis. Notably, we have found that RNF144B, via its putative ubiquitin ligase activity, could regulate proteins essential for cellular processes involved in preserving genomic stability, mitotic progression, and DNA damage. The targets identified include a known RNF144B target, p21, that prompts cell cycle arrest [34, 78], TPX2 microtubule nucleation factor required for normal assembly of mitotic spindles [79], TOP2A, a DNA topoisomerase that is required for mitotic chromosome condensation and segregation [80] and RAD21, a cohesin complex protein that regulates sister chromatid cohesion and separation [81]. Notably, we observed elevated TRP53 protein levels in non-transformed RNF144B deficient cells MEFs and to lesser extent in 3KT cells, while such elevation was not detected in A549 LUAD cancer cells with abrogated RNF144B. This suggests that TP53 regulation is likely independent of RNF144B or may occur in a tissue- or stage-specific manner. However, it is clear that the ubiquitin–proteasome pathway is one of the main factors in p53 regulation during tumor development [82, 83]. The multiple layers of negative and positive regulation governing TP53 presents challenges to understand the pathways crucial for regulating TP53 stability. Consequently, further studies are needed to validate whether RNF144B modulates TP53 expression through its E3-ligase activity in lung adenocarcinomas.

Consistent with a notion that RNF144B has a role in DNA repair [23], we have shown increased DNA double strand breaks in response to γ-IR in RNF144B-deficient cells, suggesting a potential deficiency in DNA repair. Beyond contributing to the DNA double strand break repair, RNF144B deficiency induced chromosomal instability, a clear hallmark of aggressive and refractory cancers [66, 84, 85]. RNF144B inactivation leads to substantial abnormalities during cell division, such as the presence of lagging chromosomes, DNA bridges and micronuclei, further emphasizing its significance in maintaining genomic integrity. While the precise mechanism by which RNF144B contributes to genomic stability maintenance remains to be fully elucidated, our study shows that cells lacking RNF144B have a higher proportion of cycling tetraploid cells, suggesting that the tetraploidy checkpoint could be partially abrogated.

Tetraploidy followed by aneuploidy is a frequent occurrence in *Tp53* mutant cancers [26, 73, 86–90]. Here we showed that cells with low RNF144B that present increased ploidy, DNA damage and chromosomal aberrations can survive and progress even in the presence of wild-type *Tp53*. Furthermore, analysis of the aneuploidy score indicated that lower levels of RNF144B mRNA correlated with chromosomal abnormalities in LUAD patients with wild-type TP53. This supports previous findings suggesting that TP53 may not be fully essential for maintaining a correct ploidy, and whole-genome doubling can occur even in the presence of functional TP53 [91–93]. In most of the cases reported, poor checkpoint regulation due to overexpression of specific cyclins or spindle assembly factors were the cause for the appearance of mitotic slippage and subsequent tetraploidization [91, 93–96]. Our results show that RNF144B could be involved in regulating the levels of several cell cycle and spindle assembly proteins, including TPX2 [79], MAP4 [97], BCCIP [98], CCNB2 [99], RAD21 [81] and KIF4 [100].

Our results provide preclinical evidence that dysregulation of DNA repair and mitotic fidelity caused by RNF144B deficiency enables lung cancer cells to evade the cytotoxic effects of drugs that cause CIN, DNA damage (through topoisomerase II inhibition) or mitotic alterations. Numerous reports suggest that the cellular response to cytotoxic agents is influenced by TP53 [77]. However, our findings indicate that TP53 alone may not be the sole determinant, as RNF144B deficient 3KT and A549 lung oncogenic expressing cells exhibited similar responses to cytotoxic agents, but variable levels of TP53. Thus, resistance to drugs that cause DNA damage or CIN likely arises from other factors that contribute to tolerance to DNA damage and genomic instability, such as improper cell cycle checkpoint control. However, further studies are needed to elucidate these mechanisms. This finding may have important implications for clinical practice, as the levels of RNF144B could serve as a prognostic marker and potentially identify patients who will not benefit from DNA damage or CIN-inducing therapies, specifically in *Tp53* wild-type LUAD cancers.

In summary, we show that RNF144B limits chromosomal instability and enables DNA damage response in the context of oncogene expressing cells. These multifaceted functions of RNF144B contribute significantly in maintaining genomic integrity. Importantly, RNF144B deficiency in lung adenocarcinoma cells induces their resistance to DNA damage, CIN or cell cycle based anti-cancer therapies.

### Limitations

The precise molecular mechanism and target genes involved in RNF144B-mediated maintenance of genomic stability and tumor prevention are yet to be determined. Assessment of the direct causative relationships between RNF144B and TP53 in regulating genomic stability are not defined. An additional constraint of our study was the lack of specificity of available anti-RNF144B antibodies.

## Conclusions

In conclusion, our study establishes RNF144B as a critical tumor suppressor, with particular significance in lung adenocarcinomas. Our findings suggest a potential role for RNF144B in maintaining genomic stability, potentially through the regulation of proteins associated with cell cycle progression, mitotic process, and DNA damage response. RNF144B deficiency leads to chromosomal instability and increased aneuploidy in human lung adenocarcinomas. Finally, RNF144B-deficiency can impact resistance to CIN-inducing cell cycle inhibitors, emphasizing its clinical relevance.

### Supplementary Information

**Additional file 1: Supplementary Figure 1.** Patient disease-survival by TP53 status and RNF144B expression. Probability of ten-year overall survival of cancer patients in human cancer samples with TP53 wild-type or TP53 mutant status and RNF144B low (below the median) or high expression (above the median). Cancer types: bladder (BLCA), breast (BRCA), colon (COAD), glioblastoma (GBM), head and neck (HNSC), kidney (KICH), liver (LICH), lung squamous (LUSC), pancreatic (PAAD), esophageal (ESCA), sarcoma (SARC), stomach (STAD), low grade glioma (LGG) and prostate (PRAD). Significance evaluated by log-rank test. **Supplementary Figure 2.** TP53 regulates the expression of RNF144B in diverse cellular contexts (A) qRT-PCR analysis of CDKN1A mRNA expression in TP53 proficient and TP53 deficient MEFs, A549, 3KT, HCT116 and mKLC upon 6h treatment with 10 μM Nutlin-3a relative to untreated cells of the same genotype. *N*=3-4 independent experiments for each cell line and cell variant, in duplicates or triplicates. Mean ± SEM, ***P ≤ 0.001; **P ≤ 0.01; *P ≤ 0.05. *p*-values, two-tailed unpaired t-test. (B) Western blot analysis of mKLC cell lines and derivatives showing TP53 expression upon 0, 6 or 24 h treatment with doxorubicin (0.2 μg/ml), as well as of HCT116 cell lines and derivatives upon 6h treatment with 10 μM Nutlin-3a. Probing for β-ACTIN was used as a loading control. Note that for mouse mKLC we used three independent TRP53 WT cell lines and two TRP53 deficient cell lines. The Western blots shown are from 1 independent experiment. (C) UCSC genome view of TP53 occupancy in the Rnf144b loci in human TP53 wild-type fibroblasts from Doxorubicin (Dox, light blue tracks) and Input control data (INPUT, black tracks). TP53 consensus binding site is shown, where R = A, G; Y = C, T; W = A, T, and matching bases to the TP53 consensus binding sequence are in red. Spacer between the two binding sites can be from 0 to 13 nucleotides. Below, TP53 binding sequence identified as p53BS1 (D) UCSC Genome Browser views of TRP53 ChIP-Seq peaks in Trp53^WT^ control (mock, gray tracks), Trp53^WT^ irradiated (IR, light blue tracks) or irradiated Trp53^MUT^ (IR, dark blue) mouse B cells. Y-axis represents normalized read counts at each position. (E) Rnf144b and control Cdkn1a (p21) TP53-dependent regulation in mouse and human cells. Data accessed from www.targetgenereg.org. TP53-dependent significant gene upregulation (light blue), downregulation (dark blue) and non-significant regulation (gray). **Supplementary Figure 3.** Cells deficient for Rnf144b have growth advantage in vivo. (A) qRT-PCR analysis of Rnf144b mRNA expression in MEFs transduced with indicated shRNAs and treated with 10μM Nutlin-3a for 6h. The mRNA levels were standardised to Hmbs. Expression is relative to the untreated control shRNA targeting Renilla Luciferase (shCTRL). *N*=3 independent MEF cell lines. **P ≤ 0.01; *P ≤ 0.05, two-tailed unpaired t-tests. (B) MEF 1.01 transduced with indicated shRNAs were injected subcutaneously into nude mice and tumor volume was measured over 20 days. (Left) Tumour volume (mm3) of the same genotype. (Right) Tumour weight at ethical endpoint. *N*=4-10 tumours/shRNA from one MEFs cell line (1.01). Data are presented as Mean ± SEM. ****P ≤ 0.0001, ***P ≤ 0.001; **P ≤ 0.01, two-way ANOVA or two-tailed unpaired t-test, respectively. (C) Competition in vivo assay using MEFs 1.01 cell line. (Top) shCTRL^MEF^ expressing mCherry were mixed 1:1 with shRNF144B^MEF^ or shTRP53^MEF^ expressing GFP, injected subcutaneously into nude mice and grown for 20 days. Tumours were analyzed by FACS. (Bottom) Representative FACS plots show input at day 0 (INPUT - cell population before inoculation) and tumour populations at day 20 with either mCherry/shCTRL, GFP/shRNF144B or GFP/shTRP53 labeled cells. Bar plots show the mean percentages ± SEM of cells expressing shRNF144B (green) or shTRP53 (blue) relative to all labeled cells (GFP+mCherry) in both input and tumour populations. N=6 tumours/shRNA. (D) qRT-PCR analysis of Rnf144b mRNA expression in A549 and 3KT transduced with indicated sgRNAs and treated with 10μM Nutlin-3a for 6h. The mRNA levels were standardised to Hmbs. Expression is relative to the untreated control sgRNA (sgNT). *N*=2-4 independent experiments for each cell line and cell variant, in triplicates. **P ≤ 0.01; *P ≤ 0.05, two-tailed unpaired t-tests. (E) Next generation DNA sequencing results of A549 (left) and 3KT (right) Rnf144b gene targeted cell lines derivatives showing alterations (INDELs) in the Rnf144b gene CRISPR targeted region. sgRNF144B^A549^ isogenic clone shows a 94,4% mutant genotype. sgRNF144B^3KT^ cells show a 98.6% mutant genotype. (F) Circular plots showing frequency of lesions observed in lungs of mice 5 months after intravenous injection with 3KT cells transduced with indicated sgRNAs. Representative histological pictures below. Scale bar = 100 μm (G) Western blot showing overexpression of His-tagged RNF144B in TP53 deficient A549 and 3KT (sgTP53^A549^ and sgTP53^3KT^) cells after 72h of transfection with pcDNA 3.1 construct carrying empty (OE-CTRL) or Rnf144b cDNA (OE-RNF144B). The Western blots shown are 1 independent blot from independent experiments. (H) Percentage of viable (AnnexinV- DAPI-) MEFs, A549 and 3KT cells after treatment with Doxorubicin (0.05 μg/ml or 0.2 μg/ml) or serum starvation (0% FBS) for 24h or 72 h, respectively. Data are presented as Mean ± SEM, relative to untreated cells. *N*=3-4 independent experiments for each cell line and cell variant. *P*-value **P ≤ 0.01, One-way ANOVA. **Supplementary Figure 4.** Identifying RNF144B regulated proteins. (A) Heatmaps of differentially abundant proteins in shRNF144B^MEF^ vs shCTRL^MEF^ classified in the four highly enriched pathways: cell cycle control (*N*=30 proteins), microtubule organization (*N*=14 proteins), DNA damage response (*N*=20 proteins) and chromatin remodeling (*N*=17 proteins). *N*=3 biological replicas/shRNA. (B) Western blot analysis from extracts from RNF144B-deficient and proficient MEF 1.04, A549 and 3KT cell lines probed for TP53 or β-ACTIN (loading control). (Left) Western blots shown are representative of 3-8 independent blots from independent experiments. (Right) Quantification of TP53 protein levels relative to respective controls. The protein levels were standardised to β-ACTIN. *P*-value *P ≤ 0.05, two-tailed t.student. **Supplementary Figure 5.** RNF144B deficiency leads to aneuploidy in oncogene expressing MEFs. DNA quantification (Edu incorporation plus propidium iodide FACS analysis) for profiling of more than 4N (aneuploid analysis) cells in MEFs transduced with indicated shRNAs. (Left) Representative histogram profiles. (Right) DNA content quantification for profiling more than 4N (aneuploid) cells (>G2). *N*=2-3 independent biological replicates per MEF cell line/shRNA. Data is represented by Mean ± SEM.****P ≤ 0.0001; ***P ≤ 0.001; *P ≤ 0.05, one-way ANOVA. **Supplementary Figure 6.** RNF144B deficiency triggers chromosomal instability and correlates with high aneuploidy in human tumors. (A) Centrosomes analysis. (Left) Representative pictures with staining for γ-Tubulin and DAPI in MEF 1.04 cell line. Scale bar: 10 μm (Right) Quantification of the centrosome number. Minimum of 300 cells/shRNA were analyzed in triplicates. **P ≤ 0.01, one-way ANOVA. (B) Mitotic pole analysis of MEF 1.01 cell line transduced with indicated shRNAs. (Left) Representative images of α-tubulin and DAPI staining. Scale bar: 10 μm (Right) Quantification of multipolar mitosis. *N*=200 mitoses/shRNA were analyzed in two independent experiments. Data presented as mean ± SEM, *P ≤ 0.05, One-way ANOVA. (C) Mitotic analysis. (Left) Representative anaphase images of α-tubulin and DAPI staining in MEF 1.01 cell line released after induced G2 arrest with 15 μm of RO-3306 transduced with indicated shRNAs. Arrows show lagging chromosomes. Scale bar: 10 μm. (Right) Quantification of aberrant mitosis containing lagging chromosomes or DNA bridges. Minimum of 200 mitoses (same proportion of the different phases of mitosis) per cell line were analyzed in two independent experiments. Data presented as mean ± SEM ****P ≤ 0.0001; ***P ≤ 0.001, calculated by one-way ANOVA. (D) Violin plots of the tumour aneuploidy scores in high versus low RNF144B mRNA expression in TCGA PANCAN cohort. ***P ≤ 0.001. two-tailed t-test. (E) GSEA analysis of proteins upregulated in shRNF144B^MEF^ detected in the proteomics analysis (Fig 3) compared with the CIN70 aneuploidy signature list, described in S. Carter et al. [66]. GSEA shows enrichment of aneuploidy CIN70 signature in shRNF144B^MEF^ protein expression signature. Top panel indicates the enrichment score, the bottom panel shows the ranking metrics of each gene. **Supplementary Figure 7.** RNF144B deficient lung cells are more resistant to several cytotoxic drugs. (A) In vitro growth of NLS-GFP tagged 3KT lung cells transduced with indicated sgRNAs treated for 48h with a panel of cytotoxic drugs: Palbociclib (1 μM, 3 μM), Abemaciclib (1 μM, 3 μM), Paclitaxel (5 nM, 10 nM), Docetaxel (5 nM, 20 nM), Etoposide (10 μM, 20 μM), Doxorubicin (0,05μg/ml, 0,2 μg/ml), Carboplatin (50 μM, 100 μM), RO-3306 (5 μM) and Nutlin-3a ( 20 μM). Cell growth was measured by measuring nuclear GFP signal of images acquired with the Operetta High Content Screening System in confocal mode. Cell growth was normalized to day 0 of the same genotype/well. At least 1000 cells/well were analysed in 3-5 independent experiments, in duplicates. ****P ≤0.0001; **P ≤ 0.01; *P ≤ 0.05, paired t-test student. (B) Western blot analysis of TP53 and γH2AX proteins in A549 and 3KT cell lines transduced with indicated sgRNAs after 24h treatment with Palbociclib (3 μM), Abemaciclib (3 μM), RO-3306 (5 μM), Etoposide (20 μM), Doxorubicin (0.2μg/ml), Carboplatin (50 μM), Paclitaxel (10 nM) and Docetaxel (20nM). Probing for β-ACTIN was used as a loading control. The Western blots shown are from 1 independent blot from independent experiments.

**Additional file 2: Supplementary Table S1.** p53 Mutations. Full list of patient samples from the GDC-TCGA pancancer dataset containing a TP53 mutation. Sample ID, mutation site, mutation base change, amino acid change, mutation effect, DNA variant allele fraction and corresponding TP53 status based on the criteria described.

**Additional file 3:** **Supplementary Table S2.** TargetGeneReg Human datasets with information regarding p53-dependent RNF144B expression. Contains information on cell ID, stress stimuli used, RNF144B expression and cell type.

**Additional file 4:** **Supplementary Table S3.** TargetGeneReg Mouse datasets with information regarding p53-dependent RNF144B expression. Contains information on cell ID, stress stimuli used, RNF144B expression and cell type.

**Additional file 5: Supplementary Tables S4.1 and S4.2.** Percentage of cells with mCherry or GFP fluorescence in the preinjection cellular sample or the subcutaneous tumour samples. Subcutaneous tumour samples from CTRL/RNF144B deficient (Table S4.1) or CTRL/p53 deficient (Table S4.2) MEFs were quantified by flow cytometry depending on their fluorescence signal. Represented are the percentage of mCherry+ cells (CTRL), GFP+ cells (shRNF144B or shTRP53, respectively) and the total number of fluorescent cells (mCherry and GFP cells). Negative cells were discarded as they account for mouse host cells. The percentage of GFP+ cells was calculated over the total population of detected fluorescent cells (mCherry + GFP). The preinjection sample (cellular solution before inoculation into the mice) was also quantified.

**Additional file 6:** **Supplementary Table S5.** List of significant differentially expressed proteins from shRNF144B^MEF^ versus shCTRL^MEF^ and the corresponding changes in mRNA expression. Proteomic values represented: mean Log2 protein abundance in shCTRL^MEF^ and shRNF144B^MEF^, Log2Fold Change in protein abundance of shRNF144B^MEF^ versus shCTRL^MEF^, *p*-value and unique peptides. Transcriptomics values represented: RNA TMM (Trimmed Mean of M-values) of shCTRL^MEF^ and shRNF144B^MEF^, Log2TMM of shRNF144B^MEF^ versus shCTRL^MEF^ and adjusted *p*-value.

**Additional file 7:** **Supplementary Movie 1. **Representative shRNF144B^MEF^ stained with siR-Hoechst undergoing mitosis after release from G2 arrest with RO-3306 inhibitor. Cells show to have difficulty in completing cell division by the presence of DNA bridges. Mitosis is incomplete and cells merge again after telophase.

**Additional file 8:** **Supplementary Movie 2. **Representative shRNF144B^MEF^ stained with siR-Hoechst undergoing mitosis after release from G2 arrest with RO-3306 inhibitor. A multipolar mitosis is displayed. After the cell completes metaphase, it divides into 4 daughter cells. Two of the daughter cells appear to have incomplete cytokinesis.

**Additional file 9:** **Supplementary Movie 3.** Representative shTRP53^MEF^ stained with siR-Hoechst undergoing mitosis after release from G2 arrest with RO-3306 inhibitor. Cells complete telophase with the presence of a long DNA bridge that fails to be resolved.

**Additional file 10:** **Supplementary Movie 4.** Representative shTRP53^MEF^ stained with siR-Hoechst undergoing mitosis after release from G2 arrest with RO-3306 inhibitor. Cells complete telophase with the presence of lagging chromosomes that evolve into micronuclei.

**Additional file 11:** **Supplementary Movie 5.** Representative shTRP53^MEF^ stained with siR-Hoechst undergoing mitosis after release from G2 arrest with RO-3306 inhibitor. Displayed are two multipolar mitosis, where dividing cell originates 4 daughter cells. Some of the daughter cells present DNA bridges that impede complete segregation of the cells.

## Data Availability

RNA-seq data have been deposited in the Gene Expression Omnibus (ncbi.nlm.nih.gov/geo) under accession number GSE262951. The mass spectrometry proteomics data have been deposited to the ProteomeXchange Consortium via the PRIDE partner repository with the dataset identifier PXD051459. The code used to reproduce the figures in the paper is available at https://github.com/Geriroso/RNF144b_paper_analysis. Data supporting the findings of this study are available within the paper and its Supplementary Information files. All material generated in this study are available upon request.

## Abbreviations

ABCA1: ATP Binding Cassette subfamily A member 1
APC: Allophycocyanin
BLCA: Bladder Cancer
BPE: Bovine Pituitary Extract
BRCA: Breast Cancer
BSA: Bovine Serum Albumin
Cas9: CRISPR-associated protein 9
CAV1: Caveolin-1
CCLE: Cancer Cell Line Encyclopedia
CDK1: Cyclin-Dependent Kinase 1
CDKN1A: Cyclin Dependent Kinase Inhibitor 1A (p21)
cDNA: Complementary DNA
CFP: Cyan Fluorescent Protein
CHIP: Chromatin Immunoprecipitation
CIN: Chromosomal Instability
COAD: Colon Adenocarcinoma
CRISPR: Clustered Regularly Interspaced Short Palindromic Repeats
DAB: 3,3'-Diaminobenzidine
DAPI: 4′,6-Diamidino-2-phenylindole
DDIT4: DNA Damage Inducible Transcript 4
DMEM: Dulbecco's Modified Eagle Medium
DNA-PKc: DNA-dependent protein kinase catalytic subunit
DOX: Doxorubicin
dUTP: Deoxyuridine Triphosphate
dTTP: Deoxythymidine triphosphate
EdU: 5-Ethynyl-2’-deoxyuridine
ERCC5: Excision Repair Cross-Complementing 5
ESCA: Esophageal carcinoma
FACS: Fluorescence-activated cell sorting
FANCC: FA Complementation Group C
FBS: Foetal bovine serum
Gadd45a: Growth Arrest and DNA Damage Inducible Alpha
GBM: Glioblastoma
GFP: Green Fluorescent Protein
GFR: Growth Factor Reduced
GLS2: Glutaminase 2
GO: Gene Ontology
GSEA: Gene Set Enrichment Analysis
GTEx: Genotype-Tissue Expression
hEGF: Human Epidermal Growth Factor
HMBS: Hydroxymethylbilane Synthase
HNSC: Head and Neck Squamous cell Carcinoma
H&E: Hematoxylin and Eosin
INDEL: Insertions and Deletions
iPUL: Intrapulmonar
IR: Irradiation
KD: Knock-down
KI67: Marker of Proliferation Ki-67
KICH: Kidney Chromophobe
KO: Knock-out
KRAS: Kirsten rat sarcoma viral oncogene
KSFM: Keratinocyte Serum Free Media
LC-MS: Liquid Chromatography–Mass Spectrometry
LGG: Low Grade Glioma
LIHC: Liver Hepatocellular Carcinoma
LUAD: Lung Adenocarcinoma
LUSC: Lung Squamous Cell Carcinoma
MAR: Missing at Random
MDM2: Murine Double Minute 2
MEF: Mouse Embryonic Fibroblasts
mKLC: Mouse KRAS Lung Carcinoma
MLH1: MutL Homolog 1
MMR: Mismatch repair
MNAR: Missing Not at Random
MSH2: MutS Homolog 2
MSI: Microsatellite instability
Myc: Myelocytomatosis oncogene
NLS: Nuclear Localization Signal
NT: Non-Targeting
OE: Over-expression
PAAD: Pancreatic adenocarcinoma
PANCAN: Pan-Cancer
PBS: Phosphate Buffered Saline
PCR: Polymerase Chain Reaction
PFA: Paraformaldehyde
pH3: Phospho-Histone H3
PI: Propidium Iodide
POLK: DNA Polymerase Kappa
PRAD: Prostate adenocarcinoma
P53RFP: P53-inducible RING-finger protein
P63: Tumour protein p63
P73: Tumour protein p63
p-γH2AX: Phosphorylated H2A.X Variant Histone
RBR: RING-Between-RING
RIPA: Radioimmunoprecipitation Assay Buffer
RNF144A: Ring Finger Protein 144A
RNF144B: Ring Finger Protein 144B
RPMI: Roswell Park Memorial Institute medium
RT: Room Temperature
SARC: Sarcoma
SDS-PAGE: Sodium dodecyl-sulfate polyacrylamide gel electrophoresis
SEM: Standard error of mean
sgRNA: Small guide RNA
shRNA: Short hairpin RNA
siRNA: Small interfering RNA
SNP: Single Nucleotide Polymorphism
STAD: Stomach adenocarcinoma
TCGA: The Cancer Genome Atlas program
tFA: Total Functional Aneuploidy
TIGAR: TP53 Induced Glycolysis Regulatory Phosphatase
TP53/ TRP53: Tumour Protein p53 (human/mouse)
WT: Wild Type
ZMAT3: Zinc Finger Matrin-Type 3
3KT: HBEC3-3KT cell line
