# Supplementary material for: The TP53-activated E3 ligase RNF144B is a tumour suppressor that prevents genomic instability
Source: J Exp Clin Cancer Res. 2024 Apr 29;43:127. doi: 10.1186/s13046-024-03045-4 (PMC11057071; doi:10.1186/s13046-024-03045-4)

Supplementary Figure 1

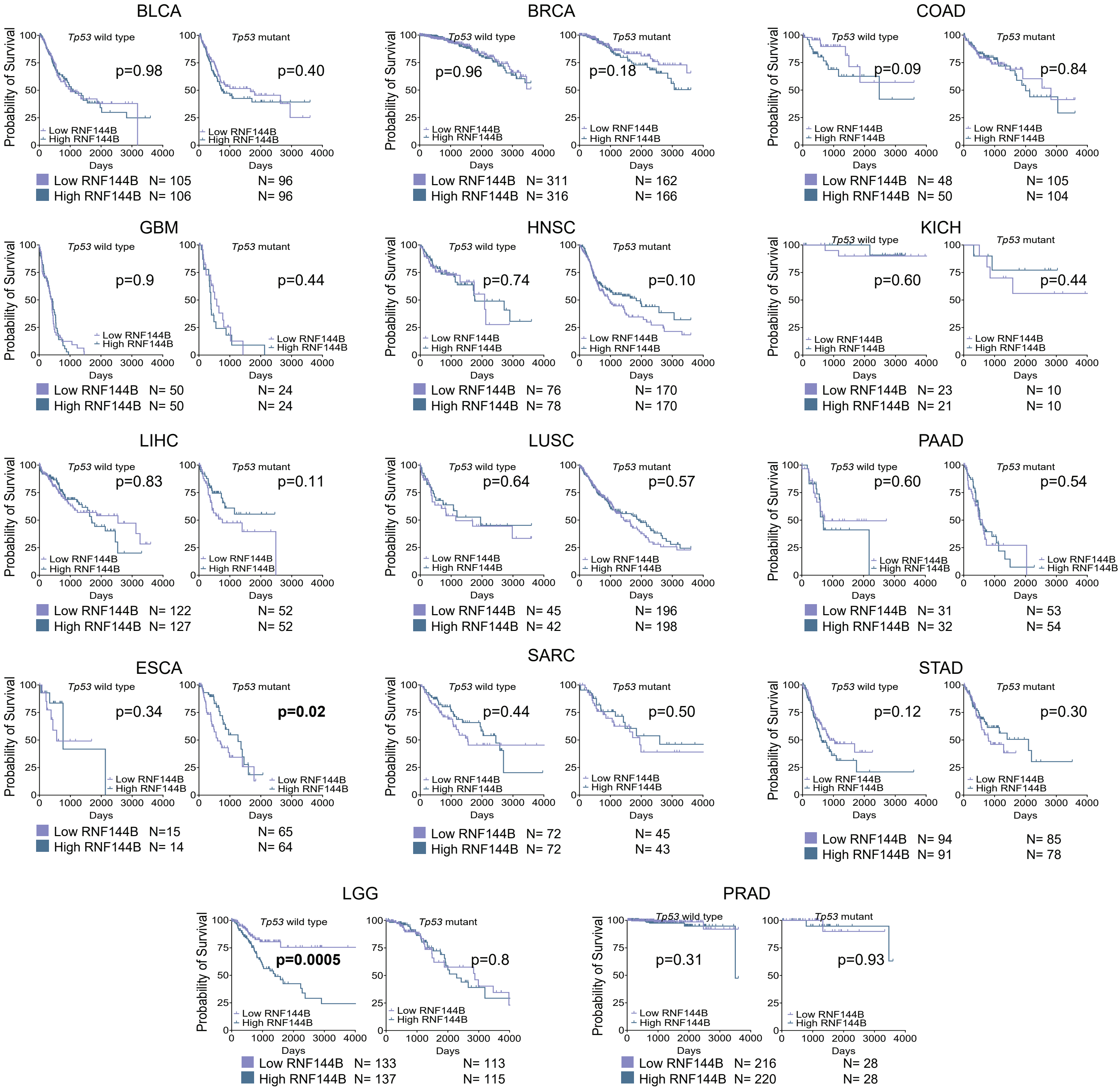

Supplementary Figure 2

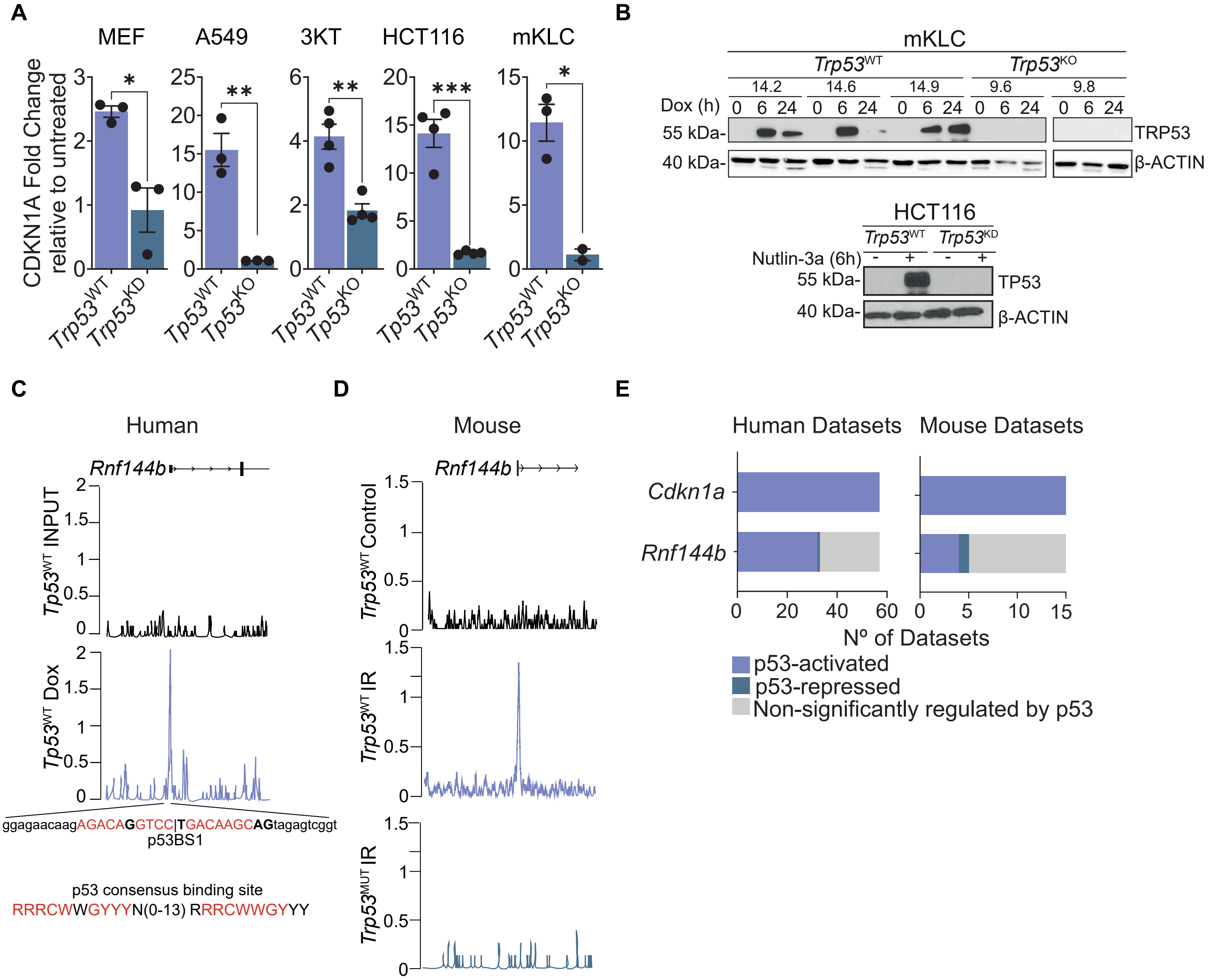

Supplementary Figure 3

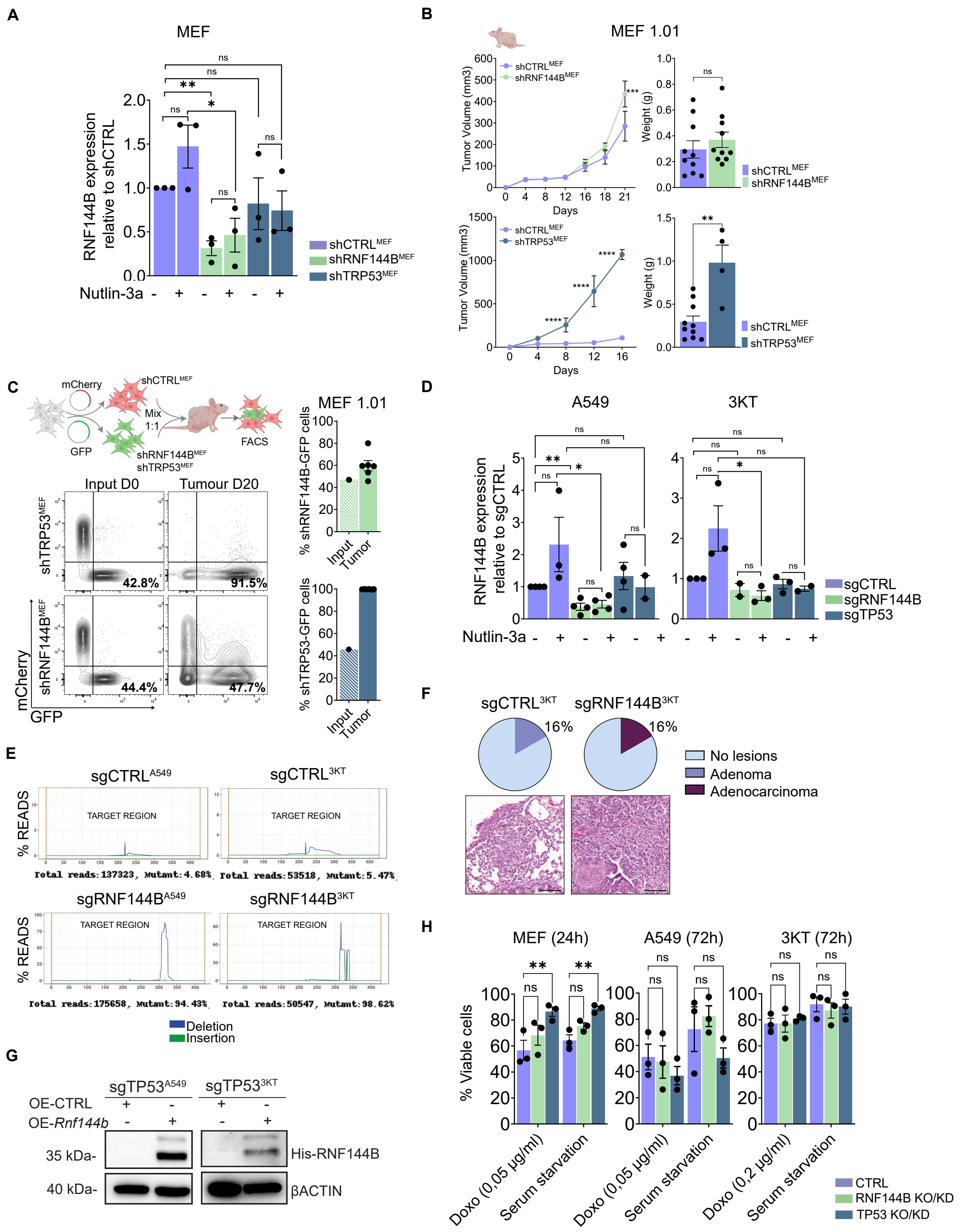

Supplementary Figure 4

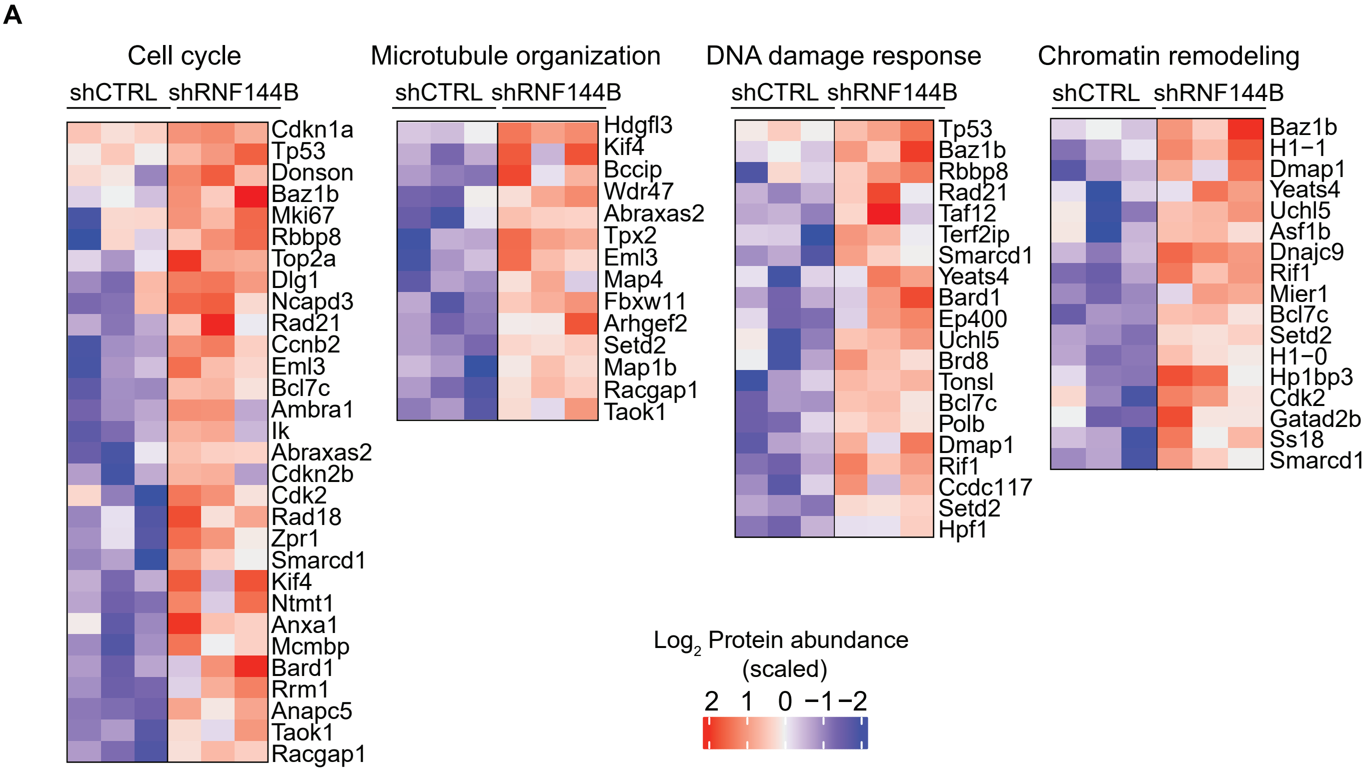

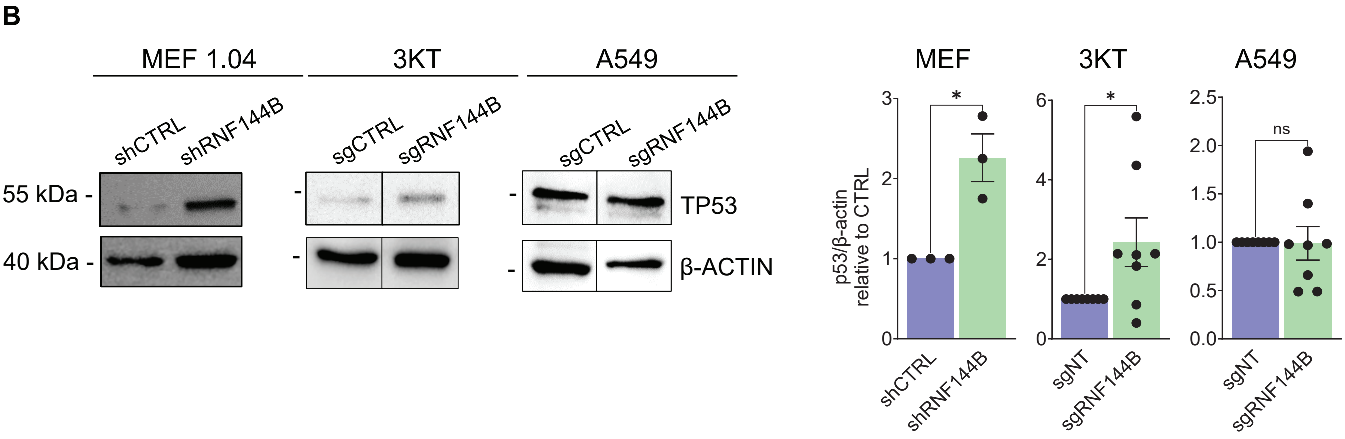

Supplementary Figure 5

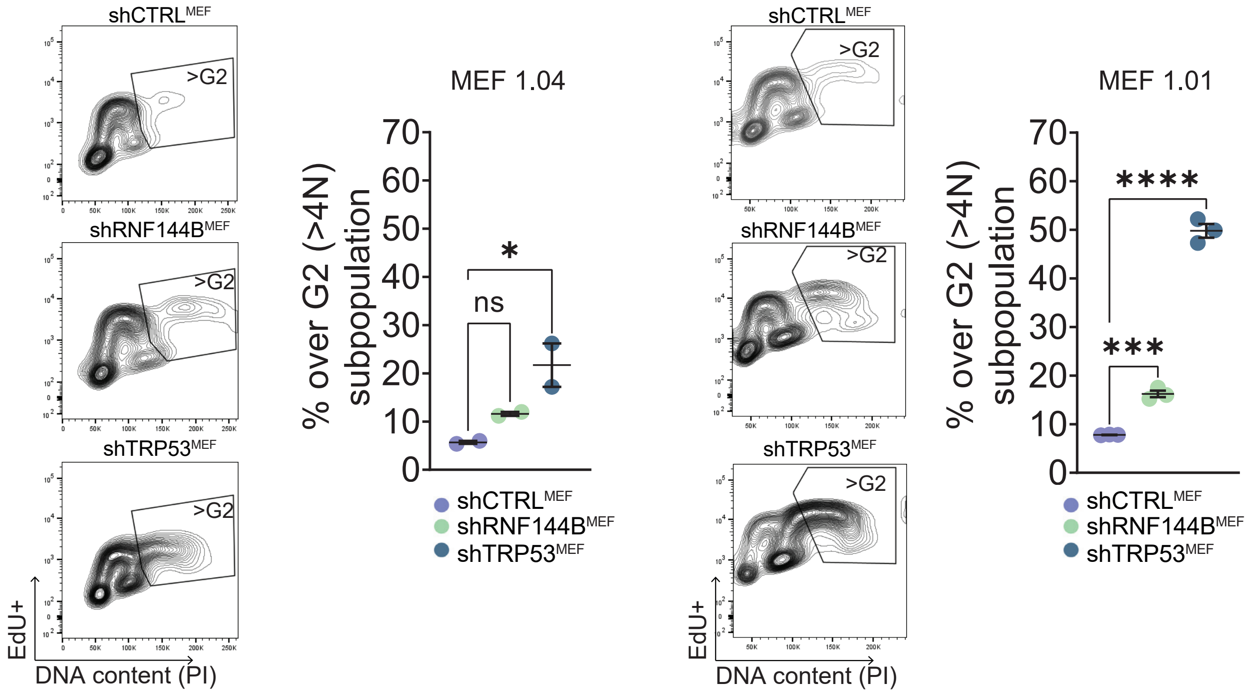

Supplementary Figure 6

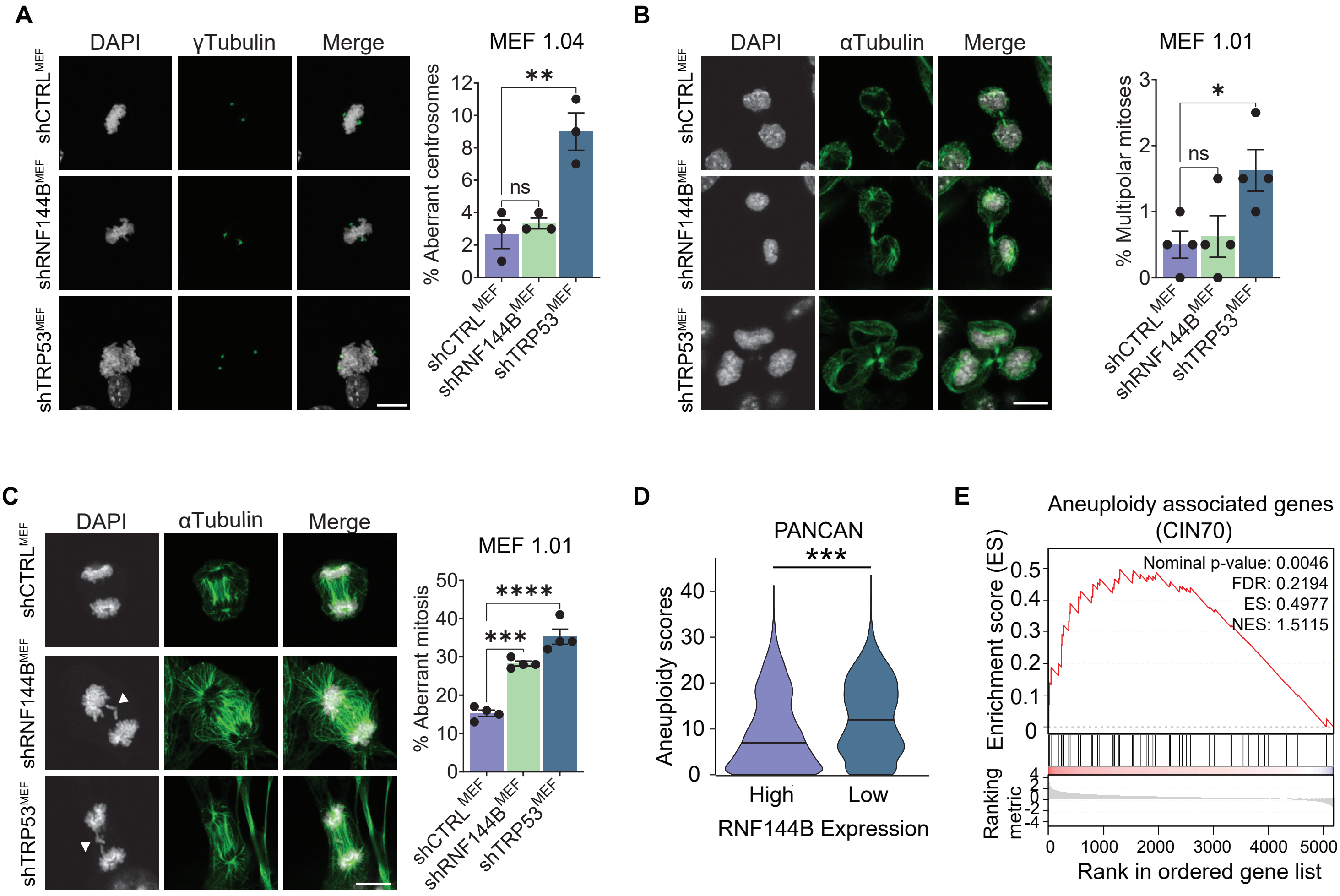

Supplementary Figure 7

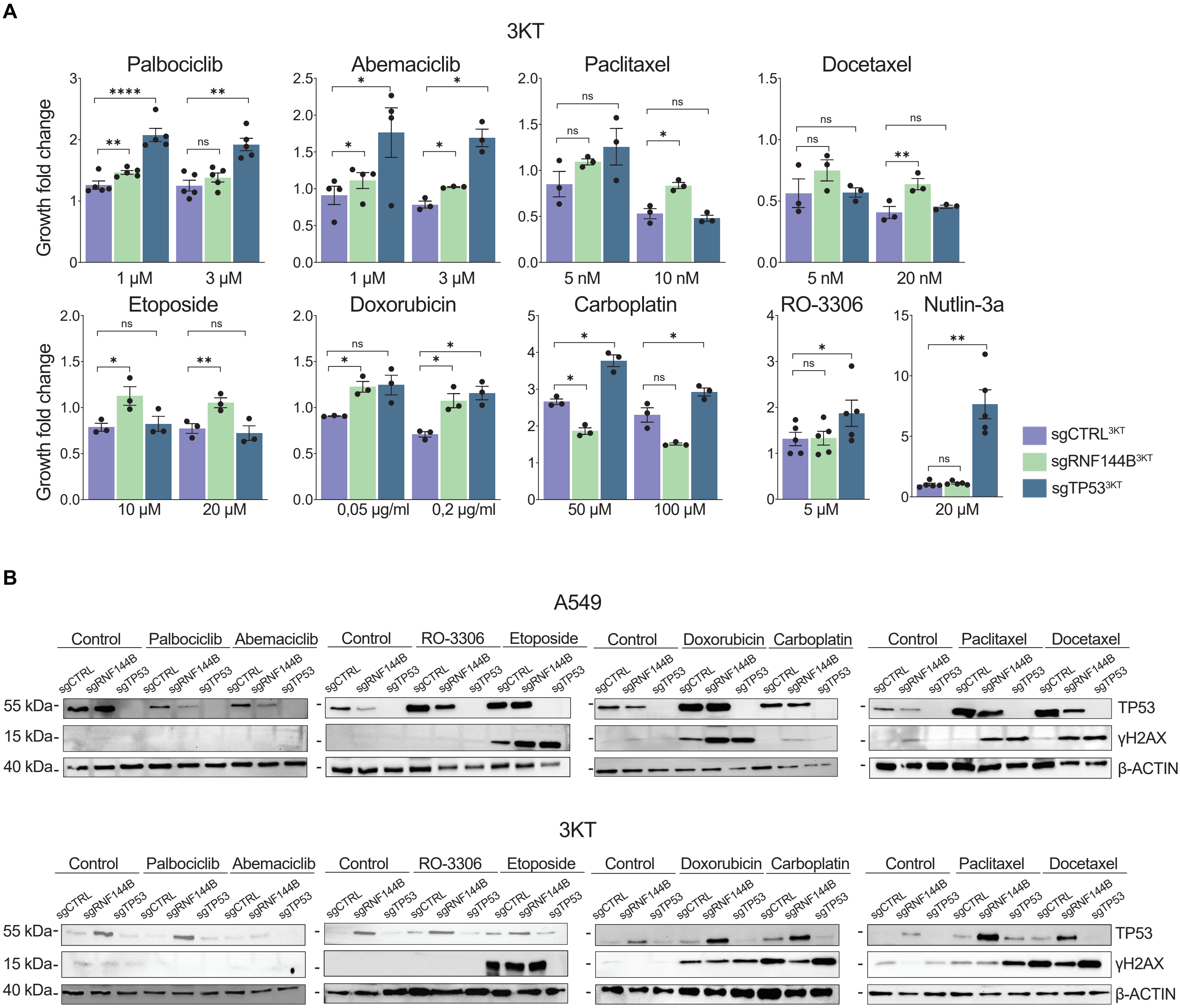

Supplement: Supplementary file 1 — Additional file 1: Supplementary Figure 1. Patient disease-survival by TP53 status and RNF144B expression. Probability of ten-year overall survival of cancer patients in human cancer samples with TP53 wild-type or TP53 mutant status and RNF144B low (below the median) or high expression (above the median). Cancer types: bladder (BLCA), breast (BRCA), colon (COAD), glioblastoma (GBM), head and neck (HNSC), kidney (KICH), liver (LICH), lung squamous (LUSC), pancreatic (PAAD), esophageal (ESCA), sarcoma (SARC), stomach (STAD), low grade glioma (LGG) and prostate (PRAD). Significance evaluated by log-rank test. Supplementary Figure 2. TP53 regulates the expression of RNF144B in diverse cellular contexts (A) qRT-PCR analysis of CDKN1A mRNA expression in TP53 proficient and TP53 deficient MEFs, A549, 3KT, HCT116 and mKLC upon 6h treatment with 10 μM Nutlin-3a relative to untreated cells of the same genotype. N=3-4 independent experiments for each cell line and cell variant, in duplicates or triplicates. Mean ± SEM, ***P ≤ 0.001; **P ≤ 0.01; *P ≤ 0.05. p-values, two-tailed unpaired t-test. (B) Western blot analysis of mKLC cell lines and derivatives showing TP53 expression upon 0, 6 or 24 h treatment with doxorubicin (0.2 μg/ml), as well as of HCT116 cell lines and derivatives upon 6h treatment with 10 μM Nutlin-3a. Probing for β-ACTIN was used as a loading control. Note that for mouse mKLC we used three independent TRP53 WT cell lines and two TRP53 deficient cell lines. The Western blots shown are from 1 independent experiment. (C) UCSC genome view of TP53 occupancy in the Rnf144b loci in human TP53 wild-type fibroblasts from Doxorubicin (Dox, light blue tracks) and Input control data (INPUT, black tracks). TP53 consensus binding site is shown, where R = A, G; Y = C, T; W = A, T, and matching bases to the TP53 consensus binding sequence are in red. Spacer between the two binding sites can be from 0 to 13 nucleotides. Below, TP53 binding sequence identified as p53BS1 (D) UC [file 13046_2024_3045_MOESM1_ESM.pdf]
